# Antimicrobial resistance of clinical Enterobacterales isolates from urine samples, Germany, 2016 to 2021

**DOI:** 10.2807/1560-7917.ES.2023.28.19.2200568

**Published:** 2023-05-11

**Authors:** Carolin Stoltidis-Claus, Kerstin Daniela Rosenberger, Falitsa Mandraka, Xenia Quante, Jörg Gielen, Dennis Hoffmann, Hilmar Wisplinghoff, Nathalie Jazmati

**Affiliations:** 1 Wisplinghoff laboratories, Cologne, Germany; 2 Institute for Medical Microbiology, Immunology and Hygiene, University of Cologne, Cologne, Germany; 3 Institute of Medical Statistics and Computational Biology, Faculty of Medicine and University Hospital Cologne, University of Cologne, Cologne, Germany; 4 Institute for Virology and Microbiology, Witten/Herdecke University, Witten, Germany

**Keywords:** AMR, urine, Enterobacterales, resistance, UTI, Germany, ID, AST, susceptibility

## Abstract

**Introduction:**

Empirical therapy for the treatment of urinary tract infections should be tailored to the current distribution and susceptibility of potential pathogens to ensure optimal treatment.

**Aim:**

We aimed to provide an up-to-date overview of the epidemiology and susceptibility of Enterobacterales isolated from urine in Germany.

**Methods:**

We retrospectively analysed antimicrobial susceptibility data from 201,152 urine specimens collected between January 2016 and June 2021 from in- and outpatients. Multiple logistic regression analysis was used to evaluate the association between year of investigation and antibiotic resistance, adjusted for age, sex and species subgroup. Subgroup analyses were performed for midstream urine samples obtained from (i) female outpatients aged 15 to 50 years, (ii) female outpatients older than 50 years and (iii) male outpatients.

**Results:**

Resistance rates of less than 20% were observed for nitroxoline (3.9%), fosfomycin (4.6%), nitrofurantoin (11.7%), cefuroxime (13.5%) and ciprofloxacin (14.2%). Resistance to trimethoprim/sulfamethoxazole (SXT) (20.1%), amoxicillin-clavulanic acid (20.5%), trimethoprim (24.2%), pivmecillinam (29.9%) and ampicillin (53.7%) was considerably higher. In the subgroup of outpatient women aged 15–50 years, resistance rates were generally lower. Resistance rates of all antibiotics decreased from 2016 to 2021. Multiple logistic regression revealed the lowest adjusted odds ratio (ORadj) of 0.838 (95% confidence interval (CI): 0.819–0.858; p < 0.001) for pivmecillinam and the highest ORadj of 0.989 (95% CI: 0.972–1.007; p = 0.226) for nitrofurantoin.

**Conclusions:**

Resistance has generally decreased over the past years, independent of sex, age and causative pathogen. Our data provide an important basis for empirical antibiotic recommendations in various settings and patient collectives.

**Key public health message**

**What did you want to address in this study?**

Knowing which antimicrobial resistances are most frequent can inform the preferred choice of antibiotic for treatment. We wanted to provide an overview of which bacteria were found in urine samples in Germany between 2016 and 2021, which antibiotics they were susceptible to and how this developed over time.

**What have we learnt from this study?**

In bacterial urinary tract infections in Germany, susceptibility has improved for all antibiotics and organisms studied, and the frequency of antibiotic resistance was lower than 20% for nitroxoline (3.9%), fosfomycin (4.6%), nitrofurantoin (11.7%), cefuroxime (13.5%) and ciprofloxacin (14.2%).

**What are the implications of your findings for public health?**

We provide important data for the assessment and future use of antimicrobial agents in empirical treatment for urinary tract infections. This up-to-date analysis of a large data collection on urinary tract infections in Germany and can serve as a basis for updating the current guidelines.

## Introduction

Urinary tract infections (UTI) are among the most common bacterial infections in Germany [1] and worldwide [2-4]; Enterobacterales, above all uropathogenic *Escherichia coli*, are the most common cause of bacterial UTI [5,6].

Because UTI are gynaecotropic women are more frequently affected than men [7]. With the exception of a (small) peak in young women aged 14−24 years, the prevalence of UTI increases with age [8] resulting in a peak in women older than 65 years [9]. Most UTI are considered to be uncomplicated UTI, defined as cystitis in a person without relevant functional or anatomical abnormalities in the urinary tract, renal dysfunctions or pre-existing or concomitant diseases that could favour an UTI or serious complications. All UTI that are not uncomplicated are considered to be complicated UTI [10].

It has proven clinically useful to divide patients with uncomplicated UTI into groups, as both the diagnostic and therapeutic approach may differ. The following major groups of patients with uncomplicated UTI can be distinguished: (i) non-pregnant premenopausal women without relevant concomitant diseases (standard group), (ii) postmenopausal women without relevant concomitant diseases, (iii) young men without relevant concomitant diseases and (iv) pregnant women without relevant concomitant diseases [10]. Furthermore, UTI can be classified into community- and hospital-acquired UTI. Hospital-acquired UTI are generally considered complicated as, among other things, the causative microorganisms are more resistant and more often are not *E. coli* [11].

Since (initial) antimicrobial therapy of UTI is usually empiric, detailed knowledge of the anticipated pathogens (in the different patient groups) and their susceptibility to antimicrobial agents is crucial. Increasing resistance against antimicrobial agents represents an important global problem with considerable challenges and costs for the healthcare system [12].

Rational use of antibiotics is essential to prevent further increase of antimicrobial resistance (AMR). Official guidelines, such as those on the management of bacterial UTI, serve as diagnostic and therapeutic orientation for practitioners in this context. In 2017, the German Association of the Scientific Medical Societies (AWMF) published an update of the interdisciplinary guideline on the management of uncomplicated bacterial community-acquired UTI [10]. This update contained first-line antibiotic recommendations for pivmecillinam (PIV), fosfomycin (FOS), nitrofurantoin (NF), nitroxoline (NIT) and, local resistance rate of less than 20% assumed, also for trimethoprim (TRI). At the same time, the use of trimethoprim/sulfamethoxazole (SXT; cotrimoxazole), cefpodoxime and fluoroquinolones in the initial antimicrobial therapy of uncomplicated UTI was firmly discouraged. The next revision of this guideline is planned for 2024.

In the present study, we aimed to describe the current resistance situation of Enterobacterales isolated from urine samples and to analyse trends over time and in different patient subgroups. We focused specifically on the patient populations discussed in the guideline in order to create the most targeted data possible for future recommendations.

## Methods

### Study design

We retrospectively analysed routine data from microbial identification (ID) and antibiotic susceptibility testing (AST) from 201,152 positive urine cultures of in- and outpatients between January 2016 and June 2021. Microbiological culture diagnostics was carried out at a large ISO 15189-accredited medical laboratory in Cologne, Germany in accordance with the applicable microbiological-infectious quality standards (MiQ) [13]. This laboratory processes urine samples from more than 4,900 outpatient practices (general medical practices and various specialist practices, including urological and gynaecological practices) and more than 100 hospitals of various sizes throughout Germany, with a focus on western Germany.

We included only urine samples yielding one pathogen and a minimum bacterial count of 10^5^ colony-forming units (cfu) per mL (n = 201,152). The variables taken into account for each sample were the patient’s age and sex, date of sample collection, type of urine specimen (i.e. midstream, catheter etc.), patient setting (outpatient, inpatient, intensive care unit, emergency room) and first digit of the postal code, species name, AST data including minimum inhibitory concentration (MIC) and/or categorial interpretation of antimicrobial testing (S/I/R) to the following antimicrobial agents: FOS, NF, NIT, PIV (only 2019–2021), ciprofloxacin (CIP), TRI, SXT, ampicillin (AMP), amoxicillin-clavulanic acid (AMC) and cefuroxime (CXM).

### Study participants

All patients with urine samples sent for cultural microbiological analysis who met the above criteria between 1 January 2016 and 30 June 2021 were included in the study. Samples were collected from outpatient and inpatient care in Germany. The dataset did not contain clinical diagnosis or comorbidities.

We defined the following subgroups of interest:

1. Subgroup S3: Premenopausal women. For this subgroup, we included data of all spontaneous midstream urine samples collected from women between 15 and 50 years of age who were seen as outpatients;
2. Subgroup S3 > 50: Postmenopausal women. This group included all spontaneous midstream urine samples from women older than 50 years who were seen as outpatients;
3. Subgroup S3men: Men. For this subgroup we included datasets from all men (all ages) that were sent from the outpatient sector and were marked as spontaneous midstream urine samples.

### Microbiology

After culture, pathogen identification was carried out by matrix-assisted laser desorption/ionisation time-of-flight mass spectrometry (MALDI-TOF MS) (BrukerDaltonics, Bremen, Germany) or the DxM MicroScan WalkAway plus system (Beckmann Coulter, Sacramento, United States (US)). The AST of Enterobacterales isolates during the study period was determined by automated microbroth dilution using the MicroScan Walkaway DxM or MicroScan WalkAway plus system (Beckmann Coulter). NIT was not included in the above automated test panels during the entire data collection period and was separately tested by disc diffusion only if specifically requested by the attending physician (mainly requested by specialised urological outpatient practices; throughout the study period, n = 1,246 NIT tests were performed and included in the analysis).

Owing to the structure of the laboratory database (archive), either MIC data and interpretation data (S/I/R) or only interpretation data (S/I/R) without MIC data were available for certain tested antibiotics. For this study, where applicable, MIC data were retrospectively interpreted consistently according to EUCAST version 10.0 for Enterobacterales to harmonise interpretation across years [14]. This was the case for AMP (resistant at MIC > 8 mg/L), PIV (resistant at MIC > 8 mg/L), CXM (resistant at MIC > 8 mg/L), FOS (resistant at MIC > 32 mg/L) and NF (resistant at MIC > 64 mg/L). We used the original AST interpretation for those substances for which the dataset did not contain MIC data. This was the case for AMC, CIP, TRI, SXT and NIT. NIT was interpreted consistently for any species since 2016 with a breakpoint of 15 mm (EUCAST for *E. coli*). Enterobacterales without individual breakpoints for NIT according to EUCAST were interpreted in analogy to *E. coli*. Intrinsically resistant bacteria were recorded as resistant according to the EUCAST guidance document *Expected resistant phenotypes version 1.1 March 2022* throughout the dataset [15].

### Statistics

Descriptive data analysis was done using IBM SPSS Statistics version 28 (IBM, Armonk, US). Continuous variables were summarised by mean and standard deviation. In the case of frequency distributions, the results are presented as simple frequency tables. Missing values were not considered for the statistical analyses. For the calculation of percentages, we excluded samples with missing values (e.g. not tested for resistance against a specific antibiotic) from the respective denominator. Thus, all percentages are based on the number of samples with available information. To investigate the association between the dependent variable (resistance to antibiotic) with various independent variables (year of investigation, age, sex, sender type, material type, pathogen group and postcode area) we carried out multiple logistic regression analysis (after dichotomisation where appropriate) for each antibiotic with simultaneous inclusion of all independent variables. Throughout the study, p values < 0.05 were considered statistically significant. Figures were created using GraphPad Prism 9 (Graphpad Software, San Diego, US).

## Results

### Study sample characteristics

The initial dataset consisted of 201,152 positive urine samples yielding only one pathogen and a minimum bacterial count of 10^5^ cfu/mL. For detailed analysis, we included only data from urine samples positive for Enterobacterales, other pathogens (n = 31,004; 15.4%) were excluded. Furthermore, cultures from the same patient and with the same causative pathogen within a period of 7 days (n = 7,880) were also excluded to avoid multiple inclusion of the same UTI.

After data preparation, 162,268 datasets of urine cultures positive for Enterobacterales obtained from in- and out-patients between 1 January 2016 and 30 June 2021 were included in the analysis. Supplementary Table S1 summarises sample and patient characteristics stratified by sex. Missing values in the variables were as follows: healthcare setting (n = 5), type of urine (n = 19), age (n = 61), postal code (n = 89), sex (n = 672) and year of sampling (no missing values).

A total of 125,363 (77.6%) of the 161,596 urine samples with information on sex were obtained from female individuals. The mean age was 67 (± 21) years, men were on average older than women (71 ± 17 vs 66 ± 22 years). With regard to urine collection, a sex difference was evident, with a greater proportion of urine samples from indwelling catheters in male individuals (males 17.2% (6,229/36,233); females 8.7% (10,931/125,363)). The majority of samples originated from north-western Germany and in particular from the postcode areas starting with the numbers 5, 4 and 2 (69.3% (111,920/161,596), 21.0% (33,976/161,596) and 8.5% (13,650/161,596), respectively). Supplementary Table S1 and Supplementary Figure S1 provide a detailed breakdown of the geographical origin of the samples.

The number of samples in subgroup S3 was 18,401 of 161,524 (11.4%). The mean age in this population was 34 (± 10) years. The number of samples in subgroup S3 > 50 was 50,244 of 161,524 (31.1%). The mean age in this population was 73 (± 11) years. The number of samples in subgroup S3men was 14,224 of 161,577 (8.8%), with a mean age of 66 (± 19) years.

### Distribution of Enterobacterales isolated from urine samples

In our initial dataset, Enterobacterales accounted for 84.6% (170,148/201,152) of the monomicrobial cultures. The most common non-Enterobacterales species were *Enterococcus faecalis* (13,857/201,152; 6.9%), *Pseudomonas aeruguinosa* (5,036/201,152; 2.5%), *Streptococcus agalactiae* (2,522/201,152; 1.3%) and *Staphylococcus aureus* (1,757/201,152; 0.9%). *Staphylococcus saprophyticus* accounted for 0.6% (1,114/201,152). Considering the Enterobacterales isolates after data preparation (n = 162,268), *E. coli* was the most commonly isolated pathogen with 67.5% (n = 109,540) followed by *Klebsiella pneumoniae* (9.7%; 15,700/162,268) and *Proteus mirabilis* (6.1% n = 9,954). For additional data on the distribution of all species found in the study period from 2016 to 2021 see Supplementary Table S2.

For a better presentation of the results, the detected species were categorised into 11 groups by genus and *E. coli* was considered separately. Table 1 shows the distribution of the four most frequent groups in relation to the patient’s sex. Supplementary Table S3 shows the data for the other, less frequent, species groups. The prevalence of extended spectrum β-lactamase (ESBL)-producing *E. coli* in our study was 5.8% (9,337/162,268). Over the years, the prevalence of ESBL-producing *E. coli* decreased steadily from 6.4% (1,276/19,962) in 2016 to 4.3% (830/19,145) in 2021. The phenomenon was similar in in- and outpatients (7.1% in 2016 (614/8,665) to 5.0% in 2021 (330/6,595) vs 5.9% (662/11,297) in 2016 to 4.0% (500/12,545) in 2021). These data differ slightly from those in Table 1 which excludes the data without information on sex.

**Table 1:** Distribution of *Escherichia* *coli*, ESBL-producing *E*. *coli*, *Klebsiella* spp. and *Proteus* spp. stratified by sex, Germany, 2016–2021 (n = 161,596)

Total n = 161,596	*E. coli* (n = 109,028)						*E. coli* (ESBL) (n = 9,308)						*Klebsiella* spp. (n = 20,352)						*Proteus* spp. (n = 10,448)					
	Female n = 91,406		Male n = 17,622		Total n = 109,028		Female n = 6,146		Male n = 2,472		Total n = 9,308		Female n = 14,185		Male n = 6,167		Total n = 20,352		Female n = 6,146		Male n = 4,302		Total n = 10,448	
	n	%	n	%	n	%	n	%	n	%	n	%	n	%	n	%	n	%	n	%	n	%	n	%
Type of urine																								
Midstream urine (n = 140,313)	83,098	74.5	15,238	53.0	98,336	70.1	5,841	5.2	1,962	6.8	7,803	5.6	12,224	11.0	4,571	15.9	16,795	12.0	4,762	4.3	2,909	10.1	7,671	5.5
Catheter urine (n = 17,160)	6,393	58.5	1,997	32.1	8,390	48.9	820	7.5	426	6.8	1,246	7.3	1,608	14.7	1,329	21.3	2,937	17.1	1171	10.7	1,158	18.6	2,329	13.6
Single catheter urine (n = 461)	315	74.8	13	32.5	328	71.1	17	4.0	6	15.0	23	5.0	51	12.1	15	37.5	66	14.3	21	5.0	1	2.5	22	4.8
Bladder puncture (n = 2,338)	1,350	70.2	146	35.2	1,496	64.0	115	6.0	22	5.3	137	5.9	228	11.9	92	22.2	320	13.7	107	5.6	69	16.6	176	7.5
Bag urine (n = 1,305)	239	47.3	225	28.1	464	35.6	40	7.9	58	7.3	98	7.5	71	14.1	160	20.0	231	17.7	85	16.8	165	20.6	250	19.2
**Total (n = 161,577)**	**91,395**	**72.9**	**17,619**	**48.6**	**109,014**	**67.5**	**6,833**	**5.5**	**2,474**	**6.8**	**9,307**	**5.8**	**14,182**	**11.3**	**6,167**	**17.0**	**20,349**	**12.6**	**6,146**	**4.9**	**4,302**	**11.9**	**10,448**	**6.5**
Healthcare setting																								
Outpatient (n = 88,543)	56,455	76.8	8,483	56.4	64,938	73.3	3,481	4.7	1,077	7.2	4,558	5.1	7,538	10.3	2,167	14.4	9,705	11.0	2,796	3.8	1,433	9.5	4,229	4.8
Emergency room	4,339	73.2	1,805	49.4	6,144	64.1	339	5.7	210	5.7	549	5.7	658	11.1	618	16.9	1,276	13.3	299	5.0	487	13.3	786	8.2
Regular ward (n = 9,688)	28,893	67.0	6,799	41.9	35,692	60.1	2,820	6.5	1082	6.7	3,902	6.6	5,555	12.9	3,103	19.1	8,658	14.6	2,821	6.5	2,208	13.6	5,029	8.5
ICU (n = 4,110)	1,719	61.9	535	40.2	2,254	54.8	194	7.0	105	7.9	299	7.3	434	15.6	279	21.0	713	17.3	230	8.3	174	13.1	404	9.8
**Total (n =161,596)**	**91,406**	**72.9**	**17,622**	**48.6**	**109,028**	**67.5**	**6,833**	**5.5**	**2,474**	**6.8**	**9,308**	**5.8**	**14,182**	**11.3**	**6,167**	**17.0**	**20,352**	**12.6**	**6,146**	**4.9**	**4,302**	**11.9**	**10,448**	**6.5**
Age groups (years)																								
0–10 (n = 3,499)	2,495	84.4	318	58.7	2,813	80.4	136	4.6	19	3.5	155	4.4	87	2.9	52	9.6	139	4.0	166	5.6	115	21.2	281	8.0
11–20 (n = 3,328)	2,551	83.0	141	55.3	2,692	80.9	110	3.6	6	2.4	116	3.5	189	6.2	54	21.2	243	7.3	127	4.1	20	7.8	147	4.4
21–30 (n = 7,384)	5,699	82.3	275	59.9	5,974	80.9	315	4.5	32	7.0	347	4.7	460	6.6	75	16.3	535	7.2	219	3.2	32	7.0	251	3.4
31–40 (n = 7,291)	5,360	81.5	462	64.4	5,822	79.9	321	4.9	46	6.4	367	5.0	482	7.3	85	11.9	567	7.8	159	2.4	38	5.3	197	2.7
41–50 (n = 8,798)	5,851	81.0	981	62.4	6,832	77.7	298	4.1	86	5.5	384	4.4	590	8.2	226	14.4	816	9.3	213	2.9	80	5.1	293	3.3
51–60 (n = 16,348)	9,275	76.5	2,409	57.1	11,684	71.5	622	5.1	266	6.3	888	5.4	1,203	9.9	659	15.6	1,862	11.4	431	3.6	315	7.5	746	4.6
61–70 (n = 23,190)	11,886	72.0	3,399	50.8	15,285	65.9	893	5.4	462	6.9	1,355	5.8	1,989	12.1	1,160	17.3	3,149	13.6	799	4.8	619	9.2	1,418	6.1
71–80 (n = 40,305)	20,729	69.9	4,899	46.1	25,628	63.6	1,625	5.5	809	7.6	2,434	6.0	4,033	13.6	1,904	17.9	5,937	14.7	1,505	5.1	1,308	12.3	2,813	7.0
81–90 (n = 42,058)	22,193	68.4	4,099	42.7	26,292	62.5	1,989	6.1	630	6.6	2,619	6.2	4,223	13.0	1,711	17.8	5,934	14.1	1,987	6.1	1,493	15.6	3,480	8.3
91–100 (n = 9,272)	5,312	68.4	613	40.6	5,925	63.9	520	6.7	115	7.6	635	6.8	922	11.9	239	15.8	1,161	12.5	534	6.9	279	18.5	813	8.8
101–110 (n = 71)	41	62.1	2	40.0	43	60.6	3	4.5	0	0.0	3	4.2	7	10.6	2	40.0	9	12.7	6	9.1	1	20.0	7	9.9
**Total (n = 161,543)**	**91,392**	**72.9**	**17,598**	**48.6**	**108,990**	**67.5**	**6,832**	**5.5**	**2,471**	**6.8**	**9,303**	**5.8**	**14,185**	**11.3**	**6,167**	**17.0**	**20,352**	**12.6**	**6,146**	**4.9**	**4,300**	**11.9**	**10,446**	**6.5**
Year of sampling																								
2016 (n = 19,851)	11,394	72.9	2,055	48.6	13,449	67.7	948	6.1	318	7.5	1,266	6.4	1,654	10.6	623	14.7	2,277	11.5	712	4.6	494	11.7	1,206	6.1
2017 (n = 25,764)	14,626	72.8	2,708	47.8	17,334	67.3	1,226	6.1	426	7.5	1,652	6.4	2,091	10.4	852	15.0	2,943	11.4	982	4.9	660	11.6	1,642	6.4
2018 (n = 28,864)	16,306	72.6	3,093	48.3	19,399	67.2	1,434	6.4	524	8.2	1,958	6.8	2,429	10.8	992	15.5	3,421	11.9	1,082	4.8	805	12.6	1,887	6.5
2019 (n = 32,764)	18,095	71.6	3,599	48.1	21,694	66.2	1,414	5.6	482	6.4	1,896	5.8	3,052	12.1	1,421	19.0	4,473	13.7	1,328	5.3	889	11.9	2,217	6.8
2020 (n = 35,247)	19,944	73.7	4,045	49.4	23,989	68.1	1,209	4.5	498	6.1	1,707	4.8	3,219	11.9	1,508	18.4	4,727	13.4	1,334	4.9	922	11.3	2,256	6.4
2021 (n = 19,106)	11,041	74.4	2,122	49.8	13,163	68.9	603	4.1	226	5.3	829	4.3	1,740	11.7	771	18.1	2,511	13.1	708	4.8	532	12.5	1240	6.5
**Total (n = 161,596)**	**91,406**	**72.9**	**17,622**	**48.6**	**109,028**	**67.5**	**6,834**	**5.5**	**2,474**	**6.8**	**9,308**	**5.8**	**14,185**	**11.3**	**6,167**	**17.0**	**20,352**	**12.6**	**6,146**	**4.9**	**4,302**	**11.9**	**10448**	**6.5**
Postal code area (first digit)																								
1 (n = 342)	237	80.3	16	34.0	253	74.0	11	3.7	6	12.8	17	5.0	20	6.8	2	4.3	22	6.4	12	4.1	10	21.3	22	6.4
2 (n = 13,650)	6,958	70.4	1,701	45.2	8,659	63.4	598	6.1	239	6.3	837	6.1	1,065	10.8	649	17.2	1,714	12.6	569	5.8	455	12.1	1,024	7.5
3 (n = 1,176)	726	74.5	117	58.2	843	71.7	43	4.4	11	5.5	54	4.6	89	9.1	31	15.4	120	10.2	59	6.1	13	6.5	72	6.1
4 (n = 33,976)	19,586	72.9	3,595	50.5	23,181	68.2	1,491	5.6	480	6.7	1,971	5.8	2,888	10.8	1,147	16.1	4,035	11.9	1,365	5.1	880	12.4	2,245	6.6
5 (n = 111,920)	63,583	73.1	12,122	48.5	75,705	67.6	4,666	5.4	1,726	6.9	6,392	5.7	10,087	11.6	4,321	17.3	14,408	12.9	4,125	4.7	2,936	11.8	7,061	6.3
6 (n = 450)	291	78.6	44	55.0	335	74.4	19	5.1	8	10.0	27	6.0	30	8.1	11	13.8	41	9.1	14	3.8	5	6.3	19	4.2
**Total (n = 161,514)**	**91,381**	**72.9**	**17,595**	**48.6**	**108,976**	**67.5**	**6,828**	**5.4**	**2,470**	**6.8**	**9,298**	**5.8**	**14,179**	**11.3**	**6,161**	**17.0**	**20,340**	**12.6**	**6,144**	**4.9**	**4,299**	**11.9**	**10,443**	**6.5**
Clinical subgroups																								
S3 (n = 18,401)	15,297	83.1	NA		15,297	83.1	721	3.9	NA		721	3.9	1,270	6.9	NA		1,270	6.9	502	2.7	NA		502	2.7
S3>50 (n = 50,244)	37,432	74.5	NA		37,432	74.5	2,507	5.0	NA		2,507	5.0	5,942	11.8	NA		5,942	11.8	1,942	3.9	NA		1,942	3.9
S3men (n = 14,224)	NA		8,279	58.2	8,279	58.2	NA		1,011	7.1	1,011	7.1	NA		2,016	14.2	2,016	14.2	NA		1,256	8.8	1,256	8.8

ESBL: extended spectrum β-lactamase; ICU: intensive care unit; NA: not applicable.

Percentage of species-specific infections within the considered stratum (female/male) of the variable of interest (listed on the left). Accordingly, it reads as follows: In midstream urine samples from female patients *E. coli* accounted for 74.5%, *Klebsiella* spp. for 11.0%, *Proteus* spp. for 4.3% and *E. coli* (ESBL) for 5.2% of all identified species (further species groups can be found in Supplementary Table S3). The denominator for the percentage calculation in this table (total number of different variables and groups divided by sex) is not shown here but can be found in Supplementary Table S1.

S3: midstream urine samples of women 15–50 years in the outpatient sector; S3 > 50: midstream urine samples of women > 50 years in the outpatient sector; S3men: midstream urine samples of men in the outpatient sector.

Depending on the origin of the sample (outpatient, emergency room, regular ward or ICU) the spectrum of pathogens varied. Thus, the percentage of *Klebsiella* spp., *Proteus* spp. and *Enterobacter* spp. infections were greater in the samples from inpatient facilities, while the percentage of *E. coli* was correspondingly lower. Compared with the overall data and S3men (67.5% (109,028/161,596) and 58.2% (8,279/14,224), respectively), *E. coli* was more prevalent in the subgroup S3 and S3 > 50 (83.1% (15,297/18,401) and 74.5% (37,432/50,244), respectively) (Table 1).

### Antimicrobial susceptibility

#### Antibiotic resistance in Enterobacterales isolated from urine samples

Missing values among the total of 162,268 were as follows: SXT (n = 41), CIP (n = 51), AMP (n = 61), NF (n = 70), AMC (n = 204), FOS (n = 224), CXM (n = 458), TRI (n = 851), PIV (n = 75,897; not tested from 2016 to 2018) and NIT (n = 161,022; only tested if requested by the attending physician).

Overall, resistance rates of NIT (3.9%; 48/1,246), FOS (4.6%, 7,378/162,044), NF (11.7%; 18,964/162,198), CXM (13.5%; 21,861/161,810) and CIP (14.2%; 22,985/162,217) were below the 20% mark for empirical therapy, whereas SXT (20.1%; 32,572/162,227), AMC (20.5%, 33,223/162,064), TRI (24.2%, 39,033/161,417), PIV (29.9%; 25,850/86,371) and AMP (53.7%; 87,155/162,207) were above it.

The resistance rates are shown as an overall rate and broken down into the individual groups of variables in Table 2.

**Table 2:** Distribution of antibiotic-specific resistances, Germany, 2016–2021 (n =162,268)

	FOS n = 162,044		NF n = 162,198		PIV n = 86,371		TRI n = 161,417		SXT n = 162,227		AMP n = 162,207		AMC n = 162,064		CXM n = 161,810		NIT n = 1,246		CIP n = 162,217	
	n (r)	%	n (r)	%	n (r)	%	n (r)	%	n (r)	%	n (r)	%	n (r)	%	n (r)	%	n (r)	%	n (r)	%
Total	7,378	4.6	18,964	11.7	25,850	29.9	39,033	24.2	32,572	20.1	87,155	53.7	33,223	20.5	21,861	13.5	48	3.9	22,985	14.2
Sex																				
Female (n = 125,363)	5,264	4.2	11,029	8.8	17,898	27.0	29,787	23.9	24,755	19.8	63,444	50.6	22,488	18.0	14,102	11.3	30	3.6	16,158	12.9
Male (n = 36,233)	2,091	5.8	7,854	21.7	7,882	40.0	9,098	25.2	7,698	21.3	23,430	64.7	10,652	29.5	7,701	21.3	18	4.5	6,770	18.7
Type of urine																				
Midstream urine (n = 140,932)	6,010	4.3	14,242	10.1	21,123	28.3	33,277	23.7	27,769	19.7	73,684	52.3	27,117	19.3	17,394	12.4	46	3.8	11,503	12.9
Catheter urine (n = 17,177)	1,124	6.5	3,907	22.8	3,952	42.0	4,637	27.2	3,870	22.5	11,001	64.1	5,050	29.5	3,702	21.6	2	10.0	1,475	15.4
Single catheter urine (n = 461)	16	3.5	42	9.1	123	26.7	125	27.2	100	21.7	239	51.8	77	16.8	43	9.3	0	0.0	9,242	15.6
Bladder puncture (n = 2,344)	132	5.6	330	14.1	384	32.9	612	26.3	510	21.8	1,314	56.1	512	21.9	364	15.6	0	0.0	765	18.6
Bag urine (n = 1,335)	96	7.2	443	33.2	266	43.6	378	28.6	319	23.9	908	68.1	466	34.9	357	26.8	0	0.0	19,012	13.5
Healthcare setting																				
Outpatient (n = 89,133)	3,501	3.9	8,026	9.0	12,895	26.1	21,319	24.0	17,766	19.9	44,260	49.7	15,052	16.9	9,385	10.6	47	3.8	3,289	19.2
Emergency room (n = 9,594)	433	4.5	1,396	14.6	1,817	31.7	2,322	24.3	1,910	19.9	5,315	55.4	2,072	21.6	1389	14.5	0	0.0	70	15.2
Regular ward (n = 59,424)	3,229	5.4	8,855	14.9	10,244	35.2	14,429	24.4	12,100	20.4	34,999	58.9	14,947	25.2	10,274	17.4	1	14.3	339	14.5
ICU (n = 4,112)	215	5.2	687	16.7	894	40.8	963	23.7	796	19.4	2,579	62.8	1,152	28.0	813	19.8	0	0.0	272	20.4
Age groups (years)																				
0–10 (n = 3,873)	65	1.7	415	10.7	535	25.0	1,015	26.3	796	20.6	1,672	43.2	572	14.8	306	7.9	0	0.0	268	6.9
11–20 (n = 3,478)	77	2.2	233	6.7	398	22.8	727	21.0	570	16.4	1,453	41.8	430	12.4	219	6.3	1	7.7	225	6.5
21–30 (n = 7,394)	201	2.7	404	5.5	849	22.5	1,661	22.6	1,290	17.4	3,256	44.0	1,081	14.6	597	8.1	1	2.4	617	8.3
31–40 (n = 7,302)	197	2.7	362	5.0	798	21.2	1,539	21.2	1,218	16.7	3,240	44.4	1,125	15.4	667	9.2	1	3.2	633	8.7
41–50 (n = 8,803)	255	2.9	586	6.7	1,029	22.5	1,847	21.1	1,514	17.2	4,096	46.5	1,400	15.9	843	9.6	1	1.1	919	10.4
51–60 (n = 16,359)	541	3.3	1,562	9.6	2,422	26.7	3,896	23.9	3,199	19.6	8,477	51.8	3,133	19.2	1,984	12.2	6	3.6	2,134	13.1
61–70 (n = 23,206)	1,025	4.4	2,753	11.9	3,889	30.5	5,625	24.4	4,780	20.6	12,735	54.9	4,913	21.2	3,242	14.0	8	4.1	3,486	15.0
71–80 (n = 40,340)	2,121	5.3	5,231	13.0	6,659	32.4	9,992	24.9	8,497	21.1	22,925	56.8	8,917	22.1	6,008	14.9	21	5.2	6,314	15.7
81–90 (n = 42,088)	2,346	5.6	6,046	14.4	7,622	33.2	10,448	25.0	8,815	21.0	24,104	57.3	9,563	22.8	6,536	15.6	8	2.9	6,816	16.2
91–100 (n = 9,293)	541	5.8	1,355	14.6	1,625	32.5	2,260	24.5	1,877	20.2	5,133	55.3	2,058	22.2	1,435	15.5	1	4.0	1,546	16.6
101–110 (n = 71)	6	8.5	13	18.3	16	42.1	9	12.7	3	4.2	31	43.7	16	22.5	12	17.4	NA		15	21.1
Years of sampling																				
2016 (n = 19,962)	1,071	5.4	2,377	11.9	NA		5,319	26.8	4,585	23.0	11,086	55.6	4,580	22.9	2,913	14.7	NA		3,489	17.5
2017 (n = 25,923)	1,263	4.9	3,041	11.7	NA		6,573	25.5	5,609	21.6	14,160	54.7	5,559	21.4	3,758	14.6	NA		4,258	16.4
2018 (n = 28,975)	1,291	4.5	3,352	11.6	NA		7,109	24.7	6,046	20.9	15,749	54.4	6,043	20.9	4,219	14.6	3	1.9	4,324	14.9
2019 (n = 32,905)	1,484	4.5	3,914	11.9	10,207	32.0	7,956	24.2	6,580	20.0	17,745	53.9	6,885	21.0	4,541	13.8	14	5.0	4,595	14.0
2020 (n = 35,358)	1,473	4.2	4,168	11.8	10,756	30.5	7,985	22.7	6,404	18.1	18,602	52.6	6,743	19.1	4,309	12.2	19	4.2	4,240	12.0
2021 (n = 19,145)	796	4.2	2,112	11.0	4,887	25.5	4,091	21.6	3,348	17.5	9,813	51.3	3,413	17.8	2,121	11.1	12	3.4	2,079	10.9
Postal code area (first digit)																				
1 (n = 343)	13	3.8	36	10.5	55	22.8	90	26.3	72	21.0	157	45.8	59	17.3	40	11.7	NA		45	13.1
2 (n = 13,658)	628	4.6	1,913	14.0	2,230	30.2	3,217	23.7	2,721	19.9	7,554	55.3	3,257	23.9	2,293	16.8	0	0.0	1,948	14.3
3 (n = 1,179)	49	4.2	117	9.9	174	26.0	320	27.2	278	23.6	632	53.6	216	18.3	121	10.3	NA		154	13.1
4 (n = 34,066)	1,646	4.8	4,049	11.9	5,343	30.1	8,580	25.3	7,248	21.3	18,503	54.3	6,965	20.5	4,561	13.4	5	3.8	4,846	14.2
5 (n = 112,477)	5,023	4.5	12,804	11.4	17,942	30.0	26,715	23.9	22,159	19.7	60,035	53.4	22,630	20.1	14,785	13.2	43	3.9	15,928	14.2
6 (n = 456)	18	3.9	35	7.7	93	22.2	96	21.1	78	17.1	215	47.1	75	16.5	47	10.3	NA		47	10.3

AMC: amoxicillin-clavulanic acid; AMP: ampicillin; CIP: ciprofloxacin; CXM: cefuroxime; FOS: fosfomycin; ICU: intensive care unit; NF: nitrofurantoin; NIT: nitroxolin; PIV: pivmecillinam; SXT: trimethoprim/sulfamethoxazole; TRI: trimethoprim; n (r): number of resistant isolates; NA: not applicable.

Number and percentage of resistant samples are given with respect to the specified antibiotic. Accordingly, it reads as follows: 4.2% of all female samples were resistant to fosfomycin, 5.8% of all male samples were resistant to fosfomycin. Missing values in the variables were as follows: sex (n = 672), type of urine (n = 19), healthcare setting (n = 5), age groups (n = 61), postal code division (n = 89). The denominator for the percentage calculation in this table is not shown here but can be found in Supplementary Table S4 for every cell of the table.

The AMR rates of the investigated antimicrobials stratified by pathogen group were calculated for the total dataset and separately for the investigated subgroups to work out and compare the clinically relevant patient groups. The data are summarised in Table 3.

**Table 3:** Distribution of resistances in different patient subgroups, stratified by species group, Germany, 2016–2021 (n = 162,268)

	Total n = 162,268		*E. coli* n = 109,540		*Klebsiella* spp. n = 20,391		*Proteus* spp. n = 10,510		*E. coli* (ESBL) n = 9,337		*Citrobacter* spp. n = 4,056		*Enterobacter* spp. n = 3,886		Other ESBL n = 1,463		*Morganella* spp. n = 1,115		*Serratia* spp. n = 1,081		*Providencia* spp. n = 344		Others n = 545		
	n (r)	%	n (r)	%	n (r)	%	n (r)	%	n (r)	%	n (r)	%	n (r)	%	n (r)	%	n (r)	%	n (r)	%	n (r)	%	n (r)	%	
Fosfomycin																									
**Total data set (n = 162,044)**	**7,378**	**4.6**	**1,252**	**1.1**	**2,544**	**12.5**	**1,243**	**11.8**	**260**	**2.8**	**80**	**2.0**	**754**	**19.4**	**270**	**18.5**	**742**	**66.8**	**55**	**5.1**	**109**	**31.8**	**69**	**12.7**	
S3 (n = 18,385)	415	2.3	149	1.0	140	11.0	45	9.0	14	1.9	3	1.1	37	18.6	10	15.4	10	52.6	3	16.7	0	0.0	4	14.8	
S3>50 (n = 50,215)	2,164	4.3	460	1.0	926	15.6	289	14.9	82	3.3	24	2.3	172	24.6	69	27.5	101	66.9	4	4.8	18	36.0	19	13.1	
S3men (n = 14,221)	665	4.7	73	0.9	185	9.2	129	10.3	29	2.9	8	1.4	69	14.8	49	23.7	95	64.6	8	4.8	15	30.0	5	12.5	
Nitrofurantoin																									
**Total data set (n = 162,198)**	**18,964**	**11.7**	**1,181**	**1.1**	**2,584**	**12.7**	**10,499**	**100.0**	**371**	**4.0**	**137**	**3.4**	**1,142**	**29.4**	**503**	**34.4**	**1,114**	**100.0**	**1,049**	**97.2**	**338**	**98.3**	**46**	**8.4**	
S3 (n = 18,390)	816	4.4	68	0.0	130	10.3	502	100.0	8	1.1	2	0.7	52	26.1	12	18.5	20	100.0	18	100.0	3	100.0	1	3.7	
S3>50 (n = 50,222)	3,919	7.8	413	1.0	868	14.6	1,938	100.0	104	4.2	34	3.3	185	26.5	86	34.3	152	100.0	80	96.4	50	100.0	9	6.2	
S3men (n = 14,223)	2,357	16.6	140	1.7	288	14.3	1,256	100.0	71	7.0	15	2.6	149	32.0	82	39.6	147	100.0	158	95.2	48	96.0	3	7.5	
Pivmecillinam																									
**Total data set (n = 86,371)**	**25,850**	**29.9**	**10,441**	**17.9**	**6,895**	**59.4**	**5,329**	**94.3**	**921**	**21.1**	**233**	**10.2**	**246**	**13.8**	**523**	**71.5**	**526**	**97.0**	**489**	**95.7**	**166**	**95.4**	**81**	**26.5**	
S3 (n = 9,617)	1,987	20.7	1,114	14.0	474	68.4	261	97.4	60	17.3	9	5.4	8	10.4	31	77.5	10	100.0	13	100.0	1	100.0	6	31.6	
S3>50 (n = 28,035)	7,080	25.3	3,467	16.6	1,925	55.7	1,048	94.1	269	21.8	46	7.3	52	15.5	99	73.9	78	96.3	43	97.7	30	96.8	23	26.4	
S3men (n = 8,269)	2,792	33.8	908	18.8	753	59.5	655	92.6	102	20.0	39	10.9	45	17.5	81	73.6	90	98.9	93	97.9	24	92.3	2	6.9	
Trimethoprim																									
**Total data set (n = 161,417)**	**39,033**	**24.2**	**24,859**	**22.8**	**2,007**	**9.9**	**4,452**	**42.8**	**5,322**	**57.6**	**287**	**7.1**	**424**	**11.0**	**1,147**	**79.3**	**299**	**26.9**	**93**	**8.7**	**62**	**18.1**	**81**	**15.9**	
S3 (n = 18,324)	3,811	20.8	3,022	19.8	93	7.4	182	36.7	433	60.2	7	2.5	8	4.0	53	81.5	4	20.0	4	22.2	0	0.0	5	20.0	
S3>50 (n = 50,005)	12,139	24.3	8,806	23.6	637	10.8	845	44.0	1,441	57.9	70	6.7	56	8.1	201	80.4	46	30.3	6	7.4	8	16.0	23	17.0	
S3men (n = 14,173)	3,594	25.4	1,797	21.8	260	12.9	593	47.5	615	61.4	31	5.3	60	12.9	166	81.0	47	32.0	13	7.9	8	16.0	4	10.0	
Trimethoprim/sulfamethoxazole																									
**Total data set (n = 162,227)**	**32,572**	**20.1**	**20,830**	**19.0**	**1,380**	**6.8**	**3,363**	**32.0**	**4,915**	**52.7**	**215**	**5.3**	**341**	**8.8**	**1,109**	**75.9**	**256**	**23.0**	**50**	**4.6**	**44**	**12.8**	**69**	**12.7**	
S3 (n = 18,398)	2,980	16.2	2,333	15.3	79	6.2	123	24.5	376	52.1	6	2.2	8	4.0	46	70.8	2	10.0	3	16.7	0	0.0	4	14.8	
S3>50 (n = 50,236)	10,283	20.5	7,516	20.1	414	7.0	650	33.5	1,344	53.6	54	5.2	45	6.4	193	76.6	37	24.3	3	3.6	7	14.0	20	13.8	
S3men (n = 14,220)	3,057	21.5	1,538	18.6	174	8.6	482	38.4	588	58.2	22	3.8	40	8.6	162	78.6	38	25.9	4	2.4	4	8.0	5	12.5	
Ampicillin																									
**Total data set (n = 162,207)**	**87,155**	**53.7**	**41,461**	**37.9**	**20,385**	**100.0**	**3,785**	**36.1**	**9,335**	**100.0**	**4,056**	**100.0**	**3,783**	**97.3**	**1,463**	**100.0**	**1,114**	**100.0**	**1,062**	**98.2**	**336**	**97.7**	**375**	**68.9**	
S3 (n = 18,395)	7,705	41.9	4,986	32.6	1,269	100.0	135	26.9	720	100.0	278	100.0	193	96.5	65	100.0	20	100.0	18	100.0	3	100.0	18	66.7	
S3>50 (n = 50,234)	24,982	49.7	13,504	36.1	5,940	100.0	703	36.2	2,506	100.0	1,040	100.0	674	96.4	252	100.0	152	100.0	81	97.6	48	96.0	82	56.6	
S3men (n = 14,217)	8,468	59.6	3,309	40.0	2016	100.0	506	40.3	1,011	100.0	586	100.0	455	97.6	207	100.0	146	100.0	162	97.6	48	96.0	22	55.0	
Amoxicillin-clavulanic acid																									
**Total data set (n = 162,064)**	**33,223**	**20.5**	**10,797**	**9.9**	**2,737**	**13.5**	**698**	**6.6**	**9,335**	**100.0**	**1,601**	**39.6**	**3,861**	**99.4**	**1,463**	**100.0**	**1,115**	**100.0**	**1,071**	**99.1**	**344**	**100.0**	**201**	**36.9**	
S3 (n = 18,389)	2,354	12.8	1,073	7.0	170	13.4	17	3.4	721	100.0	63	22.7	198	99.0	65	100.0	20	100.0	18	100.0	3	100.0	6	22.2	
S3>50 (n = 50,187)	8,054	16.0	3,147	8.4	569	9.6	102	5.3	2,507	100.0	460	44.2	695	99.4	252	100.0	152	100.0	82	98.8	50	100.0	38	26.2	
S3men (n = 14,204)	3,478	24.5	899	10.9	301	15.0	84	6.7	1,011	100.0	139	23.9	462	99.4	207	100.0	147	100.0	164	98.8	50	100.0	14	35.0	
Cefuroxime																									
**Total data set (n = 161,810)**	**21,861**	**13.5**	**3,063**	**2.8**	**1,944**	**9.6**	**760**	**7.2**	**9,332**	**100.0**	**886**	**21.9**	**2,138**	**55.1**	**1,462**	**100.0**	**1,006**	**90.3**	**1,066**	**98.8**	**71**	**20.6**	**133**	**24.5**	
S3 (n = 18,357)	1,250	6.8	238	1.6	50	4.0	12	2.4	720	100.0	43	15.6	85	42.7	65	100.0	18	90.0	18	100.0	0	0.0	1	3.7	
S3>50 (n = 50,133)	4,906	9.8	912	2.4	395	6.7	112	5.8	2,505	100.0	161	15.5	317	45.4	251	100.0	139	92.1	82	98.0	7	14.0	25	17.4	
S3men (n = 141,80)	2,467	17.0	274	3.3	221	11.0	97	7.7	1,011	100.0	97	16.6	251	54.0	207	100.0	128	87.1	165	99.4	7	14.0	9	23.1	
Nitroxoline																									
**Total data set (n = 1,246)**	**48**	**3.9**	**10**	**1.2**	**22**	**11.5**	**6**	**14.0**	**0**	**0.0**	**3**	**9.4**	**3**	**9.4**	**1**	**20.0**	**0**	**0.0**	**2**	**28.6**	**1**	**33.3**	**0**		**0.0**
S3 (n = 141)	2	1.4	1	0.8	0	0.0	1	50.0	0	0.0	0	0.0	0	0.0	NA		NA		0	0.0	NA		NA		
S3>50 (n = 667)	27	4.0	6	1.3	13	14.0	3	13.6	0	0.0	1	6.7	3	27.3	0	0.0	0	0.0	0	0.0	1	50.0	0		0.0
S3men (n = 389)	16	4.1	3	1.4	8	9.3	0	0.0	0	0.0	2	12.5	0	0.0	1	50.0	0	0.0	2	40.0	NA		0		0.0
Ciprofloxacin																									
**Total data set (n = 162,217)**	**22,985**	**14.2**	**12,516**	**11.4**	**902**	**4.4**	**1,694**	**16.1**	**6,126**	**65.7**	**206**	**5.1**	**265**	**6.8**	**926**	**63.3**	**151**	**13.6**	**109**	**10.1**	**42**	**12.2**	**48**	**8.8**	
S3 (n = 18,397)	1,383	7.5	937	6.1	23	1.8	337	46.7	51	10.2	6	2.2	4	2.0	22	33.8	0	0.0	1	5.0	0	0.0	2	11.1	
S3>50 (n = 50,232)	6,618	13.2	4,262	11.4	188	3.2	1,585	63.3	313	16.1	46	4.4	27	3.9	149	59.1	22	14.5	13	9.0	6	12.0	7	8.4	
S3men (n = 14218)	2,677	18.8	1,202	14.5	147	7.3	299	23.8	773	76.5	24	4.1	31	6.7	144	69.6	22	13.3	24	16.3	4	10.0	7	14.0	

n (r): number of resistant isolates; S3: midstream urine samples of women 15–50 years in the outpatient sector; S3 > 50: midstream urine samples of women > 50 years in the outpatient sector; S3men: midstream urine samples of men in the outpatient sector; NA: not applicable.

Number and percentage of resistant isolates are given with respect to the indicated species group. Accordingly, it reads as follows: 1.1% of all *E. coli* were resistant to fosfomycin in the total dataset, 1.0% of all *E. coli* in the subgroup S3 were resistant to fosfomycin.

The denominator for the percentage calculation in this table is not shown here but can be found in Supplementary Table S5 for every cell of the table. The total number of values taken into account for analysis of the different antibiotics are given in brackets.

Susceptibilities were highest for all antibiotics in the subgroup S3, followed by S3 > 50 and S3men (Table 3). Interestingly, this was also seen within a species. For example, susceptibility to CIP in *E. coli* was 93.9% (14,357/15,294) in S3 but 85.5% (7,073/8,275) in S3men. Fosfomycin resistance in *Klebsiella* spp. was lower in the subgroup of male outpatients (S3men: 9.2%; 185/2,015) compared with the female (outpatients) subgroups (15.6% (926/5,937) in S3 > 50 and 11.0% (140/1,268) in S3).

#### Trends in antibiotic resistance from 2016 to 2021

**Figure 1:**
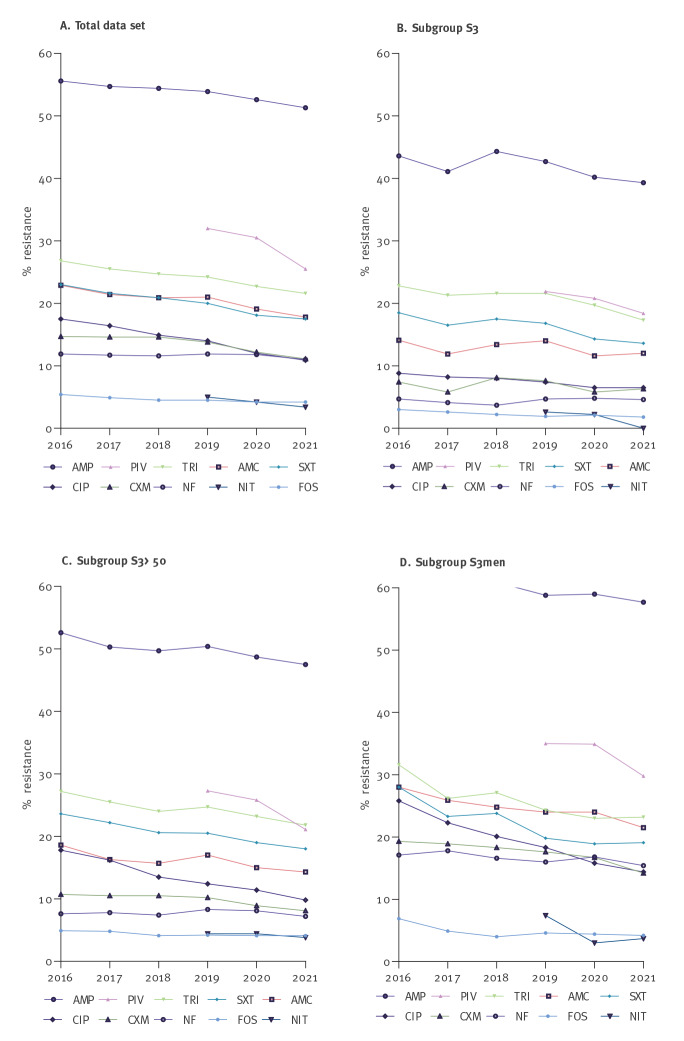
Percentage of antimicrobial resistance per year, Germany, 2016–2021 (n = 162,268)

AMC: amoxicillin-clavulanic acid; AMP: ampicillin; CIP: ciprofloxacin; CXM: cefuroxime; FOS: fosfomycin; NF: nitrofurantoin; NIT: nitroxoline (only 2019 – 2021); PIV: pivmecillinam (only 2019–2021); SXT: trimethoprim/sulfamethoxazole; TRI: trimethoprim.

S3: midstream urine samples of women 15–50 years in the outpatient sector; S3 > 50: midstream urine samples of women > 50 years in the outpatient sector; S3men: midstream urine samples of men in the outpatient sector.

Over the study period, resistance decreased for all antibiotics when considering the overall dataset and also in the studied subgroups (Figure 1).

A multiple logistic regression analysis of each antibiotic adjusted for sex, age, origin, type of urine, pathogen group and postcode area was performed to find out whether the described decrease over the years was independent of other potentially influencing variables in the overall dataset. See Table 4 for the first-line-antibiotics PIV, NF, FOS, TRI and Supplementary Table S6 for other antibiotics AMP, AMC, CXM, SXT and CIP.

**Table 4:** Multiple logistic regression models evaluating the association between sample characteristics and resistance to four first-line antibiotics, Germany, 2016–2021 (n = 162,268)

	Pivmecillinam			Fosfomycin			Trimethoprim			Nitrofurantoin		
	ORadj	95% CI	p value	ORadj	95% CI	p value	ORadj	95% CI	p value	ORadj	95% CI	p value
Study year	0.838	0.819–0.858	<0.001	0.944	0.929–0.959	<0.001	0.953	0.945–0.960	<0.001	0.989	0.972–1.007	0.226
Sex male	1.179	1.130–1.229	<0.001	0.712	0.671–0.755	<0.001	1.042	1.012–1.074	0.007	1.219	1.147–1.296	<0.001
Age (years)	1.002	1.001–1.003	<0.001	1.008	1.006–1.009	<0.001	1.002	1.002–1.003	<0.001	1.007	1.005–1.009	<0.001
Type of urine	·		0.047	·		0.334	·		<0.001	·		<0.001
Midstream urine	Reference			Reference			Reference			Reference		
Catheter urine	1.082	1.018–1.149	0.011	1.021	0.945–1.104	0.597	1.177	1.128–1.227	<0.001	1.276	1.172–1.389	<0.001
Single catheter urine	1.041	0.822–1.320	0.737	0.917	0.547–1.540	0.744	1.355	1.093–1.679	0.006	1.228	0.753–2.002	0.410
Bladder puncture urine	1.100	0.947–1.279	0.211	1.182	0.971–1.440	0.096	1.191	1.078–1.317	0.001	1.244	1.003–1.543	0.047
Bag urine	0.869	0.697–1.082	0.209	0.873	0.692–1.100	0.249	1.180	1.033–1.347	0.015	1.478	1.170–1.867	0.001
Healthcare setting	·		<0.001	·		<0.001	·		<0.001	·		<0.001
Outpatient	Reference			Reference			Reference			Reference		
Emergency rooms	1.014	0.941–1.093	0.714	0.873	0.778–0.980	0.022	0.930	0.881–0.983	0.010	0.902	0.800–1.017	0.093
Regular ward	1.185	1.137–1.235	<0.001	0.886	0.836–0.939	<0.001	0.921	0.895–0.948	<0.001	0.779	0.730–0.831	<0.001
ICU	1.348	1.205–1.508	<0.001	0.797	0.680–0.934	0.005	0.833	0.767–0.905	<0.001	0.670	0.563–0.797	<0.001
Pathogen	·		<0.001	·		<0.001	·		<0.001	·		<0.001
*E. coli*	Reference			Reference			Reference			Reference		
*E. coli* (ESBL)	1.155	1.070–1.247	<0.001	2.495	2.179–2.858	<0.001	4.507	4.313–4.709	<0.001	3.618	3.210–4.077	<0.001
Other ESBL	10.641	9.034–12.534	<0.001	21.114	18.261–24.414	<0.001	12.564	11.048–14.289	<0.001	44.392	39.196–50.276	<0.001
*Klebsiella* spp.	6.405	6.132–6.690	<0.001	12.643	11.782–13.567	<0.001	0.365	0.348–0.384	<0.001	12.618	11.743–13.558	<0.001
*Enterobacter* spp.	0.653	0.569–0.749	<0.001	22.253	20.148–24.579	<0.001	0.400	0.361–0.443	<0.001	35.451	32.319–38.887	<0.001
*Serratia* spp.	90.607	59.021–139.097	<0.001	5.507	4.165–7.282	<0.001	0.306	0.246–0.379	<0.001	2,991.7	2,055.9–4,353.6	<0.001
*Citrobacter* spp.	0.492	0.429–0.565	<0.001	1.854	1.475–2.331	<0.001	0.255	0.226–0.287	<0.001	2.984	2.491–3.575	<0.001
*Proteus* spp.	70.171	62.565–78.701	<0.001	12.313	11.325–13.388	<0.001	2.465	2.362–2.572	<0.001	Excluded^a^ (n = 10,510)		
*Morganella* spp.	136.350	82.836–224.437	<0.001	189.839	164.960–218.472	<0.001	1.202	1.051–1.375	0,007	Excluded^a^ (n = 1,115)		
*Providencia* spp.	87.540	42.991–178.252	<0.001	46.001	36.263–58.353	<0.001	0.702	0.533–0.925	0.012	4,562.6	2,029.4–10,257.8	<0.001
Others	1.587	1.229-2.050	<0.001	12.888	9.941–16.708	<0.001	0.640	0.505–0.813	<0.001	8.087	5.942–11.007	<0.001
Postal code area (first digit)	·		<0.001	·		0.116	·		<0.001	·		0.019
Postcode area 5	Reference			Reference			Reference			Reference		
Postcode area 2	0.839	0.786–0.894	<0.001	0.939	0.856–1.031	0.185	0.973	0.930–1.018	0.230	1.009	0.913–1.116	0.856
Postcode area 3	0.882	0.713–1.089	0.243	0.980	0.718–1.337	0.899	1.230	1.074–1.407	0.003	0.753	0.514–1.102	0.144
Postcode area 4	1.056	1.011–1.103	0.015	1.076	1.011–1.145	0,021	1.074	1.042–1.106	<0.001	1.111	1.038–1.19	0.002
Postcode area 1	0.943	0.663–1.339	0.742	1.234	0.683–2.229	0.487	1.180	0.917–1.518	0.198	0.965	0.479–1.946	0.921
Postcode area 6	0.922	0.707–1.204	0.553	1.155	0.691–1.931	0.583	0.844	0.664–1.072	0.165	0.661	0.334–1.308	0.235

CI: confidence interval; ESBL: extended-spectrum β-lactamase; ICU: intensive care unit; OR: odds ratio.

^a^ Very high intrinsic resistance rates in this species group did not allow the regression model to converge. Species groups with resistance rates of 100% to a specific antibiotic were therefore excluded from the respective regression analysis.

Multiple logistic regression model: Variable(s) entered: study year, sex, age, type of urine, healthcare setting, species groups, postcode area.

**Figure 2:**
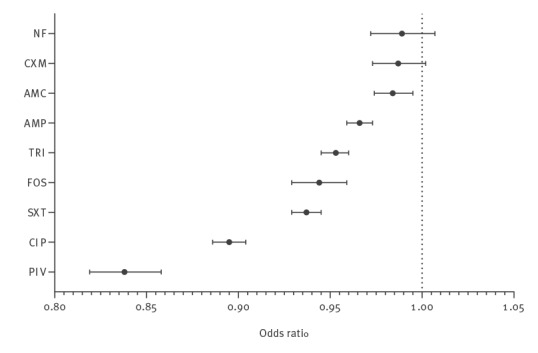
Comparison of the risk of development of antimicrobial resistance over the years (adjusted odds ratios), Germany, 2016–2021 (n = 162,268)

AMP: ampicillin; AMC: amoxicillin-clavulanic acid; CIP: ciprofloxacin; CXM: cefuroxime; FOS: fosfomycin; NF: nitrofurantoin; PIV: pivmecillinam; SXT: trimethoprim/sulfamethoxazole; TRI: trimethoprim.

The resistance rates decreased significantly for the antibiotics PIV (−6,5% from 2019 (32.0%) to 2021 (25.5%), adjusted odds ratio (ORadj) = 0.838; p < 0.001), CIP (−6,6% from 2016 (17.5%) to 2021 (10.9%), ORadj = 0.895; p < 0.001), SXT (−5.5% from 2016 (23.0%) to 2021 (17.5%), ORadj = 0.937; p < 0.001), TRI (−5.2% from 2016 (26.8%) to 2021 (21.6%), ORadj = 0.935; p < 0.001), AMC (−5.1% from 2016 (22.9%) to 2021 (17.8%), ORadj = 0.984; p = 0.004), AMP (−4.3% from 2016 (55.6%) to 2021 (51.3%), ORadj = 0.966 p < 0.001) and FOS (−1.2% from 2016 (5.4%) to 2021 (4.2%), ORadj = 0.944; p < 0.001), whereas the decrease for CXM (−3.6% from 2016 (14.7) to 2021 (11.1%), ORadj = 0.987; p = 0.093) and for NF (−0.9% from 2016 (11.9%) to 2021 (11.0%), ORadj = 0.989; p = 0.226) was not statistically significant. Because the number of resistant isolates was small, no statistically reliable development could be derived for NIT (48 resistant isolates over the whole study period), but mere descriptive statistics suggest a percentage decrease in resistant isolates also here from 2019 to 2021. Table 5 summarises the ORadj for development of antimicrobial resistance over the years and Figure 2 visualises the ORadj of the different antibiotics for a comparison.

**Table 5:** Development of antimicrobial resistance over the years Germany, 2016–2021 (n = 162,268)

	% Resistant isolates						Adjusted OR^a^	95% CI	p value
	2016	2017	2018	2019	2020	2021			
PIV	NA			32.0	30.5	25.5	0.838	0.819–0.858	<0.001
CIP	17.5	16.4	14.9	14.0	12.0	10.9	0.895	0.886–0.904	<0.001
SXT	23.0	21.6	20.9	20.0	18.1	17.5	0.937	0.929–0.945	<0.001
FOS	5.4	4.9	4.5	4.5	4.2	4.2	0.944	0.929–0.959	<0.001
TRI	26.8	25.5	24.7	24.2	22.7	21.6	0.953	0.945–0.960	<0.001
AMP	55.6	54.7	54.4	53.9	52.6	51.3	0.966	0.959–0.973	<0.001
AMC	22.9	21.4	20.9	21.0	19.1	17.8	0.984	0.974–0.995	0.004
CXM	14.7	14.6	14.6	13.8	12.2	11.1	0.987	0.973–1.002	0.093
NF	11.9	11.7	11.6	11.9	11.8	11.0	0.989	0.972–1.007	0.226
NIT	NA			5.0	4.2	3.4	Not enough resistant isolates for statistical analysis (n = 48 in total)		

AMP: ampicillin; AMC: amoxicillin-clavulanic acid; CI: confidence interval; CIP: ciprofloxacin; CXM: cefuroxime; FOS: fosfomycin; NF: nitrofurantoin; NIT: nitroxoline; OR: odds ratio; PIV: pivmecillinam; SXT: trimethoprim/sulfamethoxazole; TRI: trimethoprim.

^a^ Multiple binary logistic regression analysis was performed for each antibiotic separately to analyse the trend of the resistance rates over the time adjusted for sex, age, origin, type of urine, pathogen group and postcode area. Detailed analyses are shown in Supplementary Table S6.

Multiple binary logistic regression analysis was performed for each antibiotic separately to analyse the trend of the resistance rates over the time adjusted for sex, age, origin, type of urine, pathogen group and postcode area. Detailed analyses are shown in Table 5 and Supplementary Table S6. Dots represent the adjusted odds ratios indicating a decrease in resistance over the years for the individual antibiotic. Whiskers represent the 95% confidence intervals. Decrease in resistance was greatest for pivmecillinam. Cefuroxime and nitrofurantoin did not reach statistical significance.

In line with the general trend observed here, the resistance rates of all investigated antibiotics also decreased in ESBL-producing *E. coli* over the years (with the exception of the per se resistant β-lactam antibiotics CXM, AMP and AMC). Among all antibiotics studied, CIP showed the largest drop in resistance rate in ESBL-producing *E. coli*, from 72.1% (917/1,271) in 2016 to 59.8% (496/830) in 2021. See Supplementary Table S7 for development of the resistance rates of ESBL-producing *E. coli* over time.

## Discussion

To our knowledge, the current study analysed the largest recent dataset on Enterobacterales isolated from urine samples in Germany. The distribution of patient characteristics (sex and age) in our dataset was comparable to data from countrywide surveillance programmes, e.g. from the Netherlands [16]. Our data also verified the increasing incidence of UTI in post-menopausal women that has been described in recent studies, adding to the well-established peak of UTI in younger women [17]. The higher proportion of urine samples from women underscores the gynaecotropy of UTI [7,18].

The most common pathogen in our study was *E. coli*, accounting for 67.5% of isolated Enterobacterales, but with a notable difference between male 48.6% and female 72.5% patients. Similar rates of *E. coli* as a causative pathogen of uncomplicated UTI in women were shown in the Antimicrobial Resistance Epidemiological Survey on Cystitis (ARESC) study [19] which found *E. coli* in 76.7% of the cases and in the ECO.SENS study with 77% [20]. The differences between men and women also confirm data from sex-focused studies such as Magliano et al. where *E. coli* was found in 52.2% of the specimens from male patients 60 years and older [21].

Extensive use of oral cephalosporines and fluoroquinolones in UTI treatment has presumably contributed to the development of ESBL-based resistance to antibiotics in the past decades [22]. In our data, the overall rate of ESBL-producing *E. coli* decreased over the years, and the phenomenon was similar in males and females as well as in in- and outpatients. These data are roughly in line with data in the Surveillance Atlas of Infectious Diseases by the European Centre for Disease Prevention and Control which shows decreasing rates of third-generation-cephalosporine resistant *E. coli* for Germany (12.3% in 2017 to 9.1% in 2021) [23]. The decreasing rates may be a consequence of a decreasing use of cephalosporines and fluoroquinolones in the last years in Germany (as per data from the antimicrobial consumption surveillance of the Robert Koch Institute in Germany [24]).

Of note, for FOS, NF and NIT, according to EUCAST v.10 [14], only breakpoints for *E. coli* and/or uncomplicated UTI are available. Lacking clinical information, all our data have been interpreted using these breakpoints and can therefore only reflect the results of the in vitro testing. Overall, resistance rates in our study for NIT, FOS and NF were 3.9%, 4.6% and 11.7%, respectively. Our data regarding the susceptibility to these antibiotics are largely consistent with previously published data (NIT [25,26], NF [26], FOS [27,28]) and suggest that the (in vitro) effectiveness has not decreased in recent years.

According to the current AWMF guidelines [10], TRI is considered for empiric first-line therapy if the local resistance rate is less than 20%. In our study, overall TRI resistance was above 20% independent from the region and other variables. Even in the subgroup S3, TRI resistance was as high as 20.8% compared with 24.3% in S3 > 50 and 25.4% in S3men. In line with our data, Salm et al. recently stated a resistance rate to TRI in men of 26.6% (Germany, 2015–2020 [25]).

In our study, *E. coli* was resistant to PIV in 17.9% overall and in 14.0%, 16.6% and 18.8% in the subgroups S3, S3 > 50 and S3men, respectively. These rates are higher than in other recent publications: In similar studies in France (2017–2018) [29] and in Germany (male outpatients, 2015–2020 [25]), PIV resistance in *E. coli* was 9.5% and 9%, respectively. In the US, 6.5% of *E. coli* were resistant to mecillinam in 2018 [26]. However, since current data for women in Germany are not available, especially for the more recent years 2019 to 2021, it is unclear if the observed differences can be attributed to differences in time and patient populations or may be related to the methods used in this study. As several companies issued warnings about potentially unreliable results of PIV testing using their respective automations in 2021, we validated our results in 2021 and saw high conformity for *E. coli* (96.4%; agreement in 107/ 111 *E. coli* isolates from urine samples) between automated testing of PIV with the DxM MicroScan WalkAway plus System and disc diffusion tests, one of the reliable methods for mecillinam testing [30]. Furthermore, the total PIV resistance rate in this validation study was 18% (20/111 resistant), perfectly in line with our epidemiological data (17.9%). Accordingly, we believe that the data for *E. coli* presented here are valid despite the general technical limitation of automated testing.

We do not believe that the overall high rates of PIV resistance in our study can be explained by increasing selection pressure due to higher consumption rates. In Germany, the number of PIV prescriptions has indeed increased steadily in both the hospital (0.01 defined daily doses (DDD)/100 patient days in 2017 to 0.48 DDD/100 patient days in 2021 [24]) and outpatient sectors (116 DDD/1,000 patient days in 2017 to 2,228 DDD/1,000 patient days in 2020 [31]). However, we saw a consistent decline in PIV resistance rates in *E. coli* from 2019 (19% resistant isolates) to 2021 (14.9% resistant isolates). Nevertheless, the consumption and resistance situation of PIV should be further monitored and also investigated in further studies in order not to miss such a phenomenon.

Despite the fact that PIV, FOS, NF, NIT and TRI were named as first-line agents for the therapy of UTI and that at the same time, the use of cotrimoxazole and fluoroquinolones in the calculated initial therapy of uncomplicated UTI was firmly discouraged in 2017, we found a clear negative trend in AMR for all of these antibiotics. However, this effect was actually greater for CIP and SXT, than for the first-line agents FOS, TRI and NF. Decreasing resistance rates over time are roughly in line with available surveillance data from the national antibiotic resistance surveillance of the Robert Koch Institute [27].

A possible explanation for this trend is greater awareness among physicians and decreasing prescription of antimicrobial agents [32,33]. Focusing on the outpatient sector in Germany, prescription rates of systemic antibiotics decreased significantly by 21% from 2010 to 2018 [34]. With UTI-associated prescriptions being secondary only to those for respiratory infections [34], this decline might well be seen in our resistance data. In addition, the European Medicines Agency (EMA) and the Federal Institute for Drugs and Medical Devices (Bundesinstitut für Arzneimittel und Medizinprodukte) issued a drug safety announcement in 2018 and 2019, indicating that fluoroquinolones such as CIP should not be used for uncomplicated infections owing to the risk of serious side effects, which may also have contributed to these observations.

All antibiotics in our study showed the lowest resistance rate in the subgroup S3, followed by S3 > 50 and S3men. This disparity was to be expected and probably largely reflects the different causative pathogens with a higher proportion of (susceptible) *E. coli* in (young) women. Nevertheless, our subgroup analysis showed that even if the infection takes place by the same species, there can be significant differences in the resistance rate depending on variables such as age and sex.

Limitations of our study include the fact that the analysis was performed of Enterobacterales isolates recovered from urinary tract samples rather than from patients with confirmed clinical uncomplicated UTI. Furthermore, requesting a urine culture for an uncomplicated UTI is explicitly not recommended by current guidelines. Our subgroup data on outpatient midstream urines can therefore not be transferred to all patients with uncomplicated urinary tract infection. 

The power of this study lies in the large number of cases including a big subset of data for geriatric patients (> 80 years, n = 51,401), which allowed us to investigate pathogen–substance combinations that are hardly ever monitored. Furthermore, all data came from one laboratory and did not represent a collection of data from different investigators with possibly different microbiological methods and interpretations. The interpretation of MIC data was harmonised using EUCAST v.10 throughout the dataset for AMP, CXM, FOS, NF, and PIV [14]. For AMC, CIP, NIT, SXT and TRI, only interpretation data (S/I/R) were available. Nevertheless, the underlying MIC to interpret Enterobacterales in the different years as resistant could be analysed continuously for AMC (resistant if MIC > 8 mg/L throughout the study period), TRI (resistant if MIC > 4 mg/L throughout the study period), SXT (resistant if MIC ≥ 4 mg/L throughout the study period) and NIT (resistant if zone diameter ≥ 15 mm throughout the study period). For CIP from 2016 to 2018, the MIC was interpreted in the laboratory as resistant if > 1 mg/L. In contrast, from 2019 to 2021, the MIC was interpreted as resistant if > 0.5 mg/L, potentially leading to an underestimation of resistance rate for CIP in the years 2016 to 2018, as isolates with a MIC of 1 mg/L would not have been interpreted as resistant. Since, according to the *Antimicrobial wild type distributions of microorganisms* published by EUCAST [35], the MIC of 1 mg/L occurs in only 0.6% (94/15,667) of all *E. coli* isolates, this difference might play only a minor role but has to be considered when interpreting the CIP data. In sum, our data provide a good basis for examining trends over time without being influenced by interpretation bias.

## Conclusion

Our analysis of a large population of patients with UTI shows a general decrease in resistance over the past years, independent of sex, causative pathogen and antimicrobial agent. The data presented here provide an important basis for the guidelines on empirical treatment of UTI in Germany.

## Supplementary Data

1304000 Supplement
