# Author’s correction for Euro Surveill. 2023;28(19)

**DOI:** 10.2807/1560-7917.ES.2023.28.24.230615c

**Published:** 2023-06-15

**Authors:** Eurosurveillance editorial team

**Affiliations:** 1 European Centre for Disease Prevention and Control (ECDC), Stockholm, Sweden

In Table 3 of the article entitled ‘Antimicrobial resistance of clinical Enterobacterales isolates from urine samples, Germany, 2016 to 2021’ by Stoltidis-Claus et al., the entries for *Proteus* spp. and *E. coli* (ESBL) were swapped for ciprofloxacin in the groups S3 and S3>50 in the version originally published on 11 May 2023. At the request of the authors, this mistake was corrected on 12 June 2023. Also, the last block of Table 3 (ciprofloxacin) was missing in the pdf version of the article. This has also been corrected.

