# Supplementary material for: Antimicrobial resistance of clinical Enterobacterales isolates from urine samples, Germany, 2016 to 2021
Source: Euro Surveill. 2023 May 11;28(19):2200568. doi: 10.2807/1560-7917.ES.2023.28.19.2200568 (PMC10176829; doi:10.2807/1560-7917.ES.2023.28.19.2200568)
Supplement: Supplement [file 22-00568_Supplement_JAZMATI.pdf]

This supplementary material is hosted by *Eurosurveillance* as supporting information alongside the article **Antimicrobial resistance of clinical Enterobacterales isolates from urine samples, Germany, 2016 to 2021** on behalf of the authors, who remain responsible for the accuracy and appropriateness of the content. The same standards for ethics, copyright, attributions and permissions as for the article apply. Supplements are not edited by *Eurosurveillance* and the journal is not responsible for the maintenance of any links or email addresses provided therein.

Supplementary Table S1: Sample characteristics stratified by sex, Germany, 2016–2021 (n = 161596)

		Female (n=125363)		Male (n=36233)		Total (n=161596)*	
		n	%	n	%	n	%
**Type of urine**	Midstream urine	111567	89.0%	28746	79.3%	140313	86.8%
	Catheter urine	10931	8.7%	6229	17.2%	17160	10.6%
	Single catheter urine	421	0.3%	40	0.1%	461	0.3%
	Bladder puncture urine	1923	1.5%	415	1.1%	2338	1.4%
	Bag urine	505	0.4%	800	2.2%	1305	0.8%
**Healthcare setting**	Outpatient	73512	58.6%	15031	41.5%	88543	54.8%
	Emergency rooms	5931	4.7%	3657	10.1%	9588	5.9%
	Regular ward	43141	34.4%	16214	44.7%	59355	36.7%
	ICU	2779	2.2%	1331	3.7%	4110	2.5%
**age groups (years)**	0 to 10	2957	2.4%	542	1.5%	3499	2.2%
	11 to 20	3073	2.5%	255	0.7%	3328	2.1%
	21 to 30	6925	5.5%	459	1.3%	7384	4.6%
	31 to 40	6574	5.2%	717	2.0%	7291	4.5%
	41 to 50	7224	5.8%	1573	4.3%	8797	5.4%
	51 to 60	12130	9.7%	4218	11.7%	16348	10.1%
	61 to 70	16498	13.2%	6692	18.5%	23190	14.4%
	71 to 80	29670	23.7%	10635	29.4%	40305	25.0%
	81 to 90	32464	25.9%	9594	26.5%	42058	26.0%
	91 to 100	7763	6.2%	1509	4.2%	9272	5.7%
	101 to 110	66	0.1%	5	0.0%	71	0.0%
**Year of sampling**	2016	15626	12.5%	4225	11.7%	19851	12.3%
	2017	20094	16.0%	5670	15.6%	25764	15.9%
	2018	22460	17.9%	6404	17.7%	28864	17.9%
	2019	25283	20.2%	7481	20.6%	32764	20.3%
	2020	27059	21.6%	8188	22.6%	35247	21.8%
	2021	14841	11.8%	4265	11.8%	19106	11.8%
**Postal code area (first digit)**	1	295	0.2%	47	0.1%	342	0.2%
	2	9884	7.9%	3766	10.4%	13650	8.5%
	3	975	0.8%	201	0.6%	1176	0.7%
	4	26858	21.4%	7118	19.7%	33976	21.0%
	5	86941	69.4%	24979	69.0%	111920	69.3%
	6	370	0.3%	80	0.2%	450	0.3%
**Total**		**125363**	**100.0%**	**36233**	**100.0%**	**161596**	**100.0%**

ICU: intensive care unit

*missing values in the category sex (n=672) were not included in the description.

Missing values in the variables were as follows: type of urine n=19; age groups n=61; postal code division n=89; origin n=5.

Supplementary Figure F1: Origin of the samples by geographical region, Germany, 2016–2021 (n = 162179)

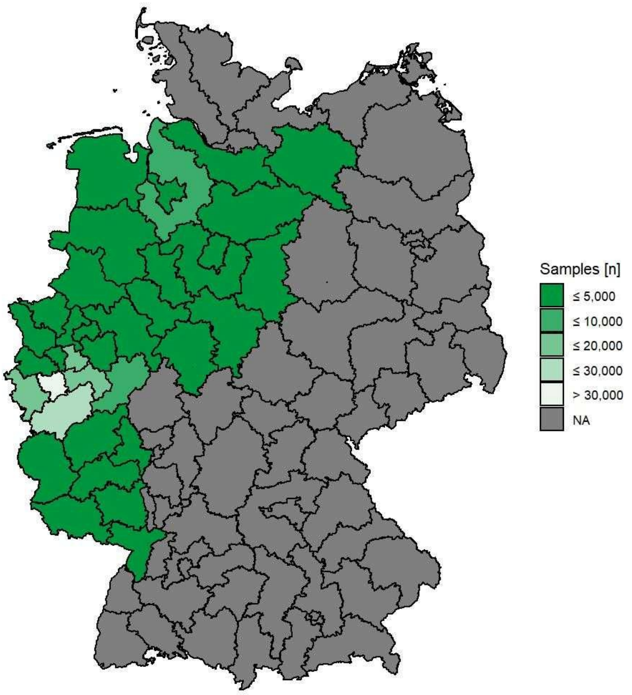

NA: No samples were sent from these areas.

Supplementary Table S2: Distribution of all species found in the study period, Germany, 2016–2021 (n = 162268)

	n	%
Escherichia coli	109540	67.5
Klebsiella pneumoniae	15700	9.7
Proteus mirabilis	9954	6.1
Escherichia coli (ESBL)	9337	5.8
Klebsiella. oxytoca	3706	2.3
Enterobacter cloacae	2748	1.7
Citrobacter koseri	2498	1.5
Klebsiella aerogenes	1499	0.9
Citrobacter freundii complex	1185	0.7
Morganella morganii	1115	0.7
Serratia marcescens	1026	0.6
Enterobacter cloacae complex	460	0.3
Proteus vulgaris	454	0.3
Providencia rettgeri	240	0.1
Citrobacter amalonaticus	118	0.1
Citrobacter farmeri	117	0.1
Raoultella ornithinolytica	116	0.1
Hafnia alvei	115	0.1
Proteus penneri	101	0.1
Providencia stuartii	94	0.1
Escherichia fergus	80	0
Enterobacter agglomerans	75	0
Klebsiella ozaenae	67	0
Citrobacter braakii	66	0
Citrobacter young	58	0
Enterobacter spp.	35	0
Klebsiella oxytoca (ESBL)	33	0
Serratia liquefaciens	33	0
Kluyvera spp.	32	0
Enterobacter asburiae	27	0
Escherichia vulnificus	16	0
Proteus mirabilis (ESBL)	16	0
Citrobacter spp.	14	0
Lellita amnigena	14	0
Salmonella typhimurim	14	0
Klebsiella variicola	12	0
Serratia spp.	12	0
Klebsiella spp.	11	0
Ewingella americana	10	0
Pantoea agglomerans	10	0
Providencia spp.	10	0
Kluyvera intermedia	9	0
Serratia odorifera	9	0
Edwardsiella tarda	8	0
Escherichia hermanii	8	0
Leclercia adecarboxylata	8	0
Proteus vulgaris (ESBL)	6	0
Enterobacter amnigenus	5	0
Enterobacter sakazakii	5	0
Cedecea davisae	4	0
Salmonella spp.	4	0
Salmonella enteritidis	3	0
Salmonella infantis	3	0
Cedecea spp.	2	0
Cronobacter sakazakii	2	0
Escherichia albertii	2	0
Enterobacterales (no species level identification)	2	0
Klebsiella rhinoscleromatis	2	0
Pantoea spp.	2	0
Proteus alcalifaciens	2	0
Ewingella spp.	1	0
Proteus hauseri	1	0
Raoultella planticola	1	0
Salmonella muenchen	1	0
Salmonella Vvrchow	1	0
Serratia plymuthica	1	0
Total	**162268**	**100**

Supplementary Table S3: Distribution of less frequent species groups stratified by sex, Germany, 2016–2021 (n = 161596)

		Enterobacter spp. (n=3874)						*Citrobacter spp. (n=4047)*						*other ESBL (n=1460)*						*others (n=544)*					
**Total (n=161596)**		Female (n=2159)		Male (n=1715)		Total (n=3874)		Female (n=2378)		Male (n=1669)		Total (n=4047)		Female (n=833)		Male (n=627)		Total (n=1460)		Female (n=392)		Male (n=152)		Total (n=544)	
		n	%	n	%	n	%	n	%	n	%	n	%	n	%	n	%	n	%	n	%	n	%	n	%
**Type of urine**	Midstream urine (n=140313)	1795	1.6%	1206	4.2%	3001	2.1%	2068	1.9%	1336	4.6%	3404	2.4%	658	0.6%	435	1.5%	1093	0.8%	1121	1.0%	1089	3.8%	2210	1.6%
	Catheter urine (n=17160)	287	2.6%	420	6.7%	707	4.1%	254	2.3%	276	4.4%	530	3.1%	148	1.4%	161	2.6%	309	1.8%	250	2.3%	462	7.4%	712	4.1%
	Single catheter urine (n=461)	6	1.4%	1	2.5%	7	1.5%	9	2.1%	2	5.0%	11	2.4%	1	0.2%	2	5.0%	3	0.7%	1	0.2%	0	0.0%	1	0.2%
	Bladder puncture (n=2338)	51	2.7%	34	8.2%	85	3.6%	34	1.8%	14	3.4%	48	2.1%	13	0.7%	9	2.2%	22	0.9%	25	1.3%	29	7.0%	54	2.3%
	Bag urine (n=1305)	20	4.0%	54	6.8%	74	5.7%	12	2.4%	41	5.1%	53	4.1%	13	2.6%	20	2.5%	33	2.5%	25	5.0%	77	9.6%	102	7.8%
	Total (n=161577)	2159	1.7%	1715	4.7%	3874	2.4%	2377	1.9%	1669	4.6%	4046	2.5%	833	0.7%	627	1.7%	1460	0.9%	1422	1.1%	1657	4.6%	3079	1.91%
**Healthcare setting**	Outpatient (n=88543)	963	1.3%	531	3.5%	1494	1.7%	1389	1.9%	631	4.2%	2020	2.3%	345	0.5%	237	1.6%	582	0.7%	545	0.7%	472	3.1%	1017	1.1%
	Emergency rooms	91	1.5%	152	4.2%	243	2.5%	79	1.3%	144	3.9%	223	2.3%	54	0.9%	62	1.7%	116	1.2%	72	1.2%	179	4.9%	251	2.6%
	Regular ward (n=9688)	1032	2.4%	950	5.9%	1982	3.3%	851	2.0%	849	5.2%	1700	2.9%	410	1.0%	303	1.9%	713	1.2%	759	1.8%	920	5.7%	1679	2.8%
	ICU (n=4110)	73	2.6%	82	6.2%	155	3.8%	59	2.1%	45	3.4%	104	2.5%	24	0.9%	25	1.9%	49	1.2%	46	1.7%	86	6.5%	132	3.2%
	Total (=161596)	2159	1.7%	1715	4.7%	3874	2.4%	2377	1.9%	1669	4.6%	4047	2.5%	833	0.7%	627	1.7%	1460	0.9%	1422	1.13%	1657	4.57%	3079	1.9%
**Age groups (years)**	0 to 10 (n=3499)	19	0.6%	20	3.7%	39	1.1%	27	0.9%	5	0.9%	32	0.9%	8	0.3%	5	0.9%	13	0.4%	19	0.6%	8	1.5%	27	0.8%
	11 to 20 (n=3328)	29	0.9%	10	3.9%	39	1.2%	56	1.8%	11	4.3%	67	2.0%	4	0.1%	3	1.2%	7	0.2%	7	0.2%	10	3.9%	17	0.5%
	21 to 30 (n=7384)	86	1.2%	14	3.1%	100	1.4%	90	1.3%	12	2.6%	102	1.4%	34	0.5%	6	1.3%	40	0.5%	22	0.3%	13	2.8%	35	0.5%
	31 to 40 (n=7291)	80	1.2%	21	2.9%	101	1.4%	107	1.6%	34	4.7%	141	1.9%	36	0.5%	11	1.5%	47	0.6%	29	0.4%	20	2.8%	49	0.7%
	41 to 50 (n=8798)	93	1.3%	77	4.9%	170	1.9%	107	1.5%	58	3.7%	165	1.9%	26	0.4%	20	1.3%	46	0.5%	46	0.6%	45	2.9%	91	1.0%
	51 to 60 (n=16348)	154	1.3%	158	3.7%	312	1.9%	243	2.0%	172	4.1%	415	2.5%	73	0.6%	64	1.5%	137	0.8%	129	1.1%	175	4.1%	304	1.9%
	61 to 70 (n=23190)	318	1.9%	346	5.2%	664	2.9%	333	2.0%	281	4.2%	614	2.6%	109	0.7%	133	2.0%	242	1.0%	171	1.0%	292	4.4%	463	2.0%
	71 to 80 (n=40305)	570	1.9%	503	4.7%	1073	2.7%	622	2.1%	518	4.9%	1140	2.8%	219	0.7%	184	1.7%	403	1.0%	367	1.2%	510	4.8%	877	2.2%
	81 to 90 (n=42058)	675	2.1%	497	5.2%	1172	2.8%	653	2.0%	491	5.1%	1144	2.7%	278	0.9%	177	1.8%	455	1.1%	466	1.4%	496	5.2%	962	2.3%
	91 to 100 (n=9272)	133	1.7%	69	4.6%	202	2.2%	135	1.7%	86	5.7%	221	2.4%	44	0.6%	23	1.5%	67	0.7%	163	2.1%	85	5.6%	248	2.7%
	101 to 110 (n=71)	1	1.5%	0	0.0%	1	1.4%	4	6.1%	0	0.0%	4	5.6%	1	1.5%	0	0.0%	1	1.4%	3	4.5%	0	0.0%	3	4.2%
	Total (n=161543)	2158	1.7%	1715	4.7%	3873	2.4%	2377	1.9%	1668	4.6%	4045	2.5%	832	0.7%	626	1.7%	1458	0.9%	1422	1.1%	1654	4.6%	3076	1.9%
**Year of sampling**	2016 (n=19851)	370	2.4%	219	5.2%	589	3.0%	269	1.7%	203	4.8%	472	2.4%	107	0.7%	79	1.9%	186	0.9%	172	1.1%	234	5.5%	406	2.0%
	2017 (n=25764)	418	2.1%	349	6.2%	767	3.0%	388	1.9%	261	4.6%	649	2.5%	121	0.6%	105	1.9%	226	0.9%	242	1.2%	309	5.4%	551	2.1%
	2018 (n=28864)	423	1.9%	280	4.4%	703	2.4%	361	1.6%	275	4.3%	636	2.2%	174	0.8%	140	2.2%	314	1.1%	251	1.1%	295	4.6%	546	1.9%
	2019 (n=32764)	377	1.5%	332	4.4%	709	2.2%	518	2.0%	340	4.5%	858	2.6%	186	0.7%	134	1.8%	320	1.0%	313	1.2%	284	3.8%	597	1.8%
	2020 (n=35247)	368	1.4%	364	4.4%	732	2.1%	522	1.9%	402	4.9%	924	2.6%	171	0.6%	117	1.4%	288	0.8%	292	1.1%	332	4.1%	624	1.8%
	2021 (n=19106)	203	1.4%	171	4.0%	374	2.0%	320	2.2%	188	4.4%	508	2.7%	74	0.5%	52	1.2%	126	0.7%	152	1.0%	203	4.8%	355	1.9%
	Total (n=161596)	2159	1.7%	1715	4.7%	3874	2.4%	2378	1.9%	1669	4.6%	4047	2.5%	833	0.7%	627	1.7%	1460	0.9%	1422	1.1%	1657	5%	3079	1.9%
**Postal code area (first digit)**	1 (n=342)	3	1.0%	6	12.8%	9	2.6%	8	2.7%	0	0.0%	8	2.3%	1	0.3%	3	6.4%	4	1.2%	3	1.0%	4	8.5%	7	2.0%
	2 (n=13650)	191	1.9%	163	4.3%	354	2.6%	229	2.3%	210	5.6%	439	3.2%	80	0.8%	94	2.5%	174	1.3%	194	2.0%	255	6.8%	449	3.3%
	3 (n=1176)	16	1.6%	9	4.5%	25	2.1%	26	2.7%	11	5.5%	37	3.1%	5	0.5%	2	1.0%	7	0.6%	11	1.1%	7	3.5%	18	1.5%
	4 (n=33976)	566	2.1%	318	4.5%	884	2.6%	473	1.8%	302	4.2%	775	2.3%	194	0.7%	107	1.5%	301	0.9%	295	1.1%	289	4.1%	584	1.7%
	5 (n=111920)	1378	1.6%	1218	4.9%	2596	2.3%	1634	1.9%	1139	4.6%	2773	2.5%	551	0.6%	421	1.7%	972	0.9%	917	1.1%	1096	4.4%	2013	1.8%
	6 (n=450)	5	1.4%	1	1.3%	6	1.3%	7	1.9%	5	6.3%	12	2.7%	2	0.5%	0	0.0%	2	0.4%	2	0.5%	6	7.5%	8	1.8%
	Total (n=161514)	2159	1.7%	1715	4.7%	3874	2.4%	2377	1.9%	1667	4.6%	4044	2.5%	833	0.7%	627	1.7%	1460	0.9%	1422	1.1%	1657	4.6%	3079	1.9%
**Clinical sub-groups**	S3 (n=18401)	200	1.1%	NA	NA	200	1.1%	278	1.5%	NA	NA	278	1.5%	65	0.4%	NA	NA	65	0.4%	68	0.4%	NA	NA	68	0.4%
	S3>50 (n=50244)	699	1.4%	NA	NA	699	1.4%	1040	2.1%	NA	NA	1040	2.1%	252	0.5%	NA	NA	252	0.5%	430	0.9%	NA	NA	430	0.9%
	S3men (n=14224)	NA	NA	466	3.3%	466	3.3%	NA	NA	586	4.1%	586	4.1%	NA	NA	207	1.5%	207	1.5%	NA	NA	403	2.8%	403	2.8%

ESBL: extended spectrum β-lactamase; ICU: intensive care unit; NA: not applicable.

Percentage of species-specific infections within the considered stratum (female/male) of the variable of interest (listed on the left). Accordingly, it reads as follows: In midstream urines from female patients *Enterobacter spp.* accounted for 1.6%. *Citrobacter spp.* for 1.9%. other ESBL for 0.6% and others for 1.0% of all identified species (the four most frequent species groups can be found in Table 2 in the manuscript). The denominator for the percentage calculation in this table (total number of different variables and groups divided by sex) is not shown here but can be found in Supplementary Table S1.

S3: midstream urine samples of women 15–50 years in the outpatient sector; S3 > 50: midstream urine samples of women > 50 years in the outpatient sector; S3men: midstream urine samples of men in the outpatient sector.

Supplementary Table S4: Total numbers of tests per antibiotic for the different variables (denominator for table 2), Germany, 2016–2021 (n =162268)

		FOS (n=162044)	NF (n=162198)	PIV (n=86371)	TRI (n=161417)	SXT (n=162227)	AMP (n=162207)	AMC (n=162064)	CXM (n=161810)	NIT (n=1246)	CIP (n=162217)
		n	n	n	n	n	n	n	n	n	n
**Sex**	Female (n=125363)	125195	125306	66388	124715	125335	125321	125229	125038	843	125329
	Male (n=36233)	36178	36220	19697	36032	36220	36215	36163	36103	403	36216
**Type of urine**	Midstream urine (n=140932)	140724	140871	74712	140243	140897	140877	140772	140538	1215	140891
	Catheter urine (n=17177)	17162	17168	9412	17044	17171	17173	17136	17123	20	17167
	Single catheter urine (n=461)	461	461	460	460	461	461	459	461	1	461
	Bladder puncture urine (n=2344)	2344	2344	1168	2329	2344	2343	2343	2339	6	2344
	Bag urine (n=1335)	1334	1335	610	1322	1335	1334	1335	1330	4	1335
**Healthcare setting**	Outpatient (n=89133)	89076	89094	49347	88722	89117	89107	89039	88922	1235	89106
	Emergency room (n=9594)	9585	9594	5739	9538	9594	9592	9580	9573	3	9592
	Regular ward (n=59424)	59271	59395	29087	59085	59401	59394	59332	59212	7	59402
	ICU (n=4112)	4107	4110	2193	4067	4110	4109	4108	4098	1	4112
**Age groups (years)**	0 to 10 (n=3873)	3866	3870	2136	3857	3872	3870	3870	3864	13	3871
	11 to 20 (n=3478)	3473	3476	1744	3464	3478	3475	3477	3467	13	3477
	21 to 30 (n=7394)	7384	7388	3769	7363	7393	7393	7387	7383	41	7393
	31 to 40 (n=7302)	7297	7299	3767	7267	7299	7300	7294	7284	31	7300
	41 to 50 (n=8803)	8798	8802	4573	8758	8801	8801	8797	8781	87	8800
	51 to 60 (n=16359)	16344	16354	9074	16294	16354	16352	16334	16323	165	16352
	61 to 70 (n=23206)	23174	23194	12770	23087	23199	23200	23182	23130	196	23196
	71 to 80 (n=40340)	40289	40327	20537	40124	40334	40331	40280	40217	402	40333
	81 to 90 (n=42088)	42014	42069	22925	41836	42076	42070	42034	41968	273	42072
	91 to 100 (n=9293)	9273	9287	5002	9235	9289	9283	9277	9263	25	9291
	101 to 110 (n=71)	71	71	38	71	71	71	71	69	0	71
**Year of sampling**	2016 (n=19962)	19860	19919	0	19881	19957	19953	19962	19862	0	19949
	2017 (n=25923)	25863	25911	0	25792	25913	25899	25923	25744	0	25913
	2018 (n=28975)	28933	28970	0	28789	28964	28966	28975	28842	159	28965
	2019 (n=32905)	32895	32900	31923	32821	32901	32902	32792	32882	282	32902
	2020 (n=35358)	35350	35354	35312	35173	35354	35348	35280	35343	457	35348
	2021 (n=19145)	19143	19144	19136	18961	19138	19139	19132	19137	348	19140
**Postal code area (first digit)**	1 (n=343)	343	343	241	342	343	343	342	342	0	343
	2 (n=13658)	13648	13650	7395	13567	13655	13652	13641	13618	3	13653
	3 (n=1179)	1179	1179	668	1175	1179	1179	1179	1174	0	1179
	4 (n=34066)	34038	34048	17769	33882	34060	34052	34019	33963	131	34057
	5 (n=112477)	112291	112433	59835	111912	112445	112436	112339	112168	1110	112440
	6 (n=456)	456	456	419	455	456	456	455	456	0	456

AMC: amoxicillin-clavulanic acid; AMP: ampicillin; CIP: ciprofloxacin; CXM: cefuroxime; FOS: fosfomycin; ICU: intensive care unit; NF: nitrofurantoin; NIT: nitroxolin; PIV: pivmecillinam; SXT: trimethoprim/sulfamethoxazole; TRI: trimethoprim; n (r): number of resistant isolates;

Supplementary Table S5: Total numbers of tests per antibiotic in the different patient subgroups, stratified by species group (denominator for

table 3), Germany, 2016–2021 (n = 162268)

		Total (n=162268)	*E. coli* (n=109540)	*Klebsiella spp.* (n=20391)	*Proteus spp.* (n=10510)	*E. coli (ESBL)* (n=9337)	*Citrobacter spp.* (n=4056)	*Enterobacter spp.* (n=3886)	*other ESBL* (n=1463)	*Morganella spp.* (n=1115)	*Serratia spp.* (n=1081)	*Providencia spp.* (n=344)	*Others* (n=545)
		**n**	n	n	n	n	n	n	n	n	n	n	n
**FOS**	Total data set	**162044**	109407	20353	10493	9321	4054	3880	1458	1111	1079	343	545
	S3	**18385**	15286	1268	502	720	278	199	65	19	18	3	27
	S3>50	**50215**	37415	5937	1942	2504	1038	699	251	151	83	50	145
	S3men	**14221**	8277	2015	1256	1011	586	466	207	147	166	50	40
**NF**	Total data set	**162198**	109500	20385	10499	9332	4055	3884	1461	1114	1079	344	545
	S3	**18390**	15289	1268	502	721	278	199	65	20	18	3	27
	S3>50	**50222**	37420	5941	1938	2504	1039	699	251	152	83	50	145
	S3men	**14223**	8278	2016	1256	1011	586	466	207	147	166	50	40
**PIV**	Total data set	**86371**	58422	11601	5654	4364	2277	1789	731	542	511	174	306
	S3	**9617**	7981	693	268	347	168	77	40	10	13	1	19
	S3>50	**28035**	20886	3459	1114	1235	628	336	134	81	44	31	87
	S3men	**8269**	4821	1266	707	510	357	257	110	91	95	26	29
**TRI**	Total data set	**161417**	109083	20309	10401	9244	4043	3866	1446	1111	1063	343	508
	S3	**18324**	15238	1263	496	719	278	199	65	20	18	3	25
	S3>50	**50005**	37270	5922	1922	2489	1039	695	250	152	81	50	135
	S3men	**14173**	8260	2009	1248	1001	584	465	205	147	164	50	40
**SXT**	Total data set	**162227**	109521	20384	10506	9332	4056	3884	1461	1114	1081	343	545
	S3	**18398**	15295	1269	502	721	278	200	65	20	18	3	27
	S3>50	**50236**	37426	5941	1942	2506	1040	699	252	152	83	50	145
	S3men	**14220**	8278	2015	1256	1011	586	465	206	147	166	50	40
**AMP**	Total data set	**162207**	109498	20387	10499	9335	4056	3886	1463	1114	1081	344	544
	S3	**18395**	15293	1269	502	720	278	200	65	20	18	3	27
	S3>50	**50234**	37425	5942	1940	2506	1040	699	252	152	83	50	145
	S3men	**14217**	8273	2016	1256	1011	586	466	207	146	166	50	40
**AMC**	Total data set	**162064**	109425	20326	10507	9335	4038	3885	1463	1115	1081	344	545
	S3	**18389**	15287	1268	502	721	278	200	65	20	18	3	27
	S3>50	**50187**	37391	5926	1942	2507	1040	699	252	152	83	50	145
	S3men	**14204**	8269	2011	1256	1011	582	465	207	147	166	50	40
**CXM**	Total data set	**161810**	109220	20314	10483	9332	4039	3880	1462	1114	1079	344	543
	S3	**18357**	15263	1265	501	720	276	199	65	20	18	3	27
	S3>50	**50133**	37342	5930	1940	2505	1039	698	251	151	83	50	144
	S3men	**14180**	8246	2009	1255	1011	585	465	207	147	166	50	39
**NIT**	Total data set	**1246**	855	191	43	71	32	32	5	2	7	3	5
	S3	**141**	125	6	2	3	1	3	0	0	1	0	0
	S3>50	**667**	478	93	22	39	15	11	3	1	1	2	2
	S3men	**389**	219	86	13	28	16	16	2	1	5	0	3
**CIP**	Total data set	**162217**	109508	20386	10505	9330	4056	3886	1462	1114	1081	344	545
	S3	**18397**	15294	1269	502	721	278	200	65	20	18	3	27
	S3>50	**50232**	37424	5942	1942	2503	1040	699	252	152	83	50	145
	S3men	**14218**	8275	2015	1255	1011	586	466	207	147	166	50	40

AMC: amoxicillin-clavulanic acid; AMP: ampicillin; CIP: ciprofloxacin; CXM: cefuroxime; FOS: fosfomycin; ICU: intensive care unit; NF: nitrofurantoin; NIT: nitroxolin; PIV: pivmecillinam; SXT: trimethoprim/sulfamethoxazole; TRI: trimethoprim; n (r): number of resistant isolates; S3: midstream urine samples of women 15–50 years in the outpatient sector; S3 > 50: midstream urine samples of women > 50 years in the outpatient sector; S3men: midstream urine samples of men in the outpatient sector; NA: not applicable.

Supplementary Table S6: Multiple logistic regression models evaluating the association between sample characteristics and resistance to CIP, SXT, AMP, AMC, CXM, Germany, 2016–2021 (n = 162268)

	Ciprofloxacin (CIP)				Trimethoprim/sulfamethoxazole (SXT)				Ampicillin (AMP)				Amoxicillin-clavulanic acid (AMC)				Cefuroxime (CXM)			
	ORadj	95% CI for ORadj		p-value	ORadj	95% CI for ORadj		p-value	ORadj	95% CI for ORadj		p-value	ORadj	95% CI for ORadj		p-value	ORadj	95% CI for ORadj		p-value
		Lower	Upper			Lower	Upper			Lower	Upper			Lower	Upper			Lower	Upper	
**Study year**	**0.895**	0.886	0.904	<0.001	**0.937**	0.929	0.945	<0.001	**0.966**	0.959	0.973	<0.001	**0.984**	0.974	0.995	0.004	**0.987**	0.973	1.002	0.093
**Sex male**	1.556	1.501	1.613	<0.001	1.084	1.050	1.119	<0.001	1.238	1.200	1.276	<0.001	1.312	1.260	1.365	<0.001	1.391	1.322	1.464	<0.001
**Age**	1.013	1.012	1.014	<0.001	1.004	1.003	1.004	<0.001	1.003	1.002	1.003	<0.001	1.003	1.002	1.004	<0.001	1.007	1.006	1.009	<0.001
**Type of urine**				<0.001				<0.001				<0.001				<0.001				<0.001
Midstream urine		Reference				Reference				Reference				Reference				Reference		
Catheter urine	1.331	1.265	1.401	<0.001	1.172	1.121	1.226	<0.001	1.176	1.125	1.229	<0.001	1.239	1.172	1.310	<0.001	1.299	1.213	1.391	<0.001
Single catheter urine	1.509	1.143	1.992	<0.001	1.310	1.040	1.651	0.022	1.090	0.876	1.356	0.438	1.077	0.782	1.483	0.649	0.803	0.476	1.357	0.413
Bladder puncture urine	1.138	0.999	1.298	<0.001	1.205	1.084	1.341	0.001	1.065	0.963	1.179	0.221	1.014	0.876	1.173	0.853	1.284	1.070	1.540	0.007
Bag urine	1.469	1.258	1.716	<0.001	1.211	1.053	1.393	0.007	1.252	1.078	1.453	0.003	1.364	1.144	1.627	0.001	1.533	1.263	1.859	<0.001
**Healthcare setting**				<0.001				<0.001				<0.001				<0.001				<0.001
Outpatient		Reference				Reference				Reference				Reference				Reference		
Emergency rooms	0.980	0.916	1.050	0.567	0.909	0.858	0.964	0.001	1.066	1.011	1.124	0.019	1.112	1.031	1.200	0.006	1.056	0.952	1.172	0.303
Regular ward	0.922	0.889	0.956	<0.001	0.911	0.884	0.939	<0.001	1.142	1.110	1.174	<0.001	1.352	1.299	1.406	<0.001	1.337	1.267	1.412	<0.001
ICU	1.073	0.974	1.181	0.152	0.810	0.741	0.884	<0.001	1.182	1.088	1.284	<0.001	1.445	1.303	1.603	<0.001	1.435	1.259	1.637	<0.001
**Pathogen**				<0.001				<0.001				<0.001				<0.001				<0.001
E. coli		Reference				Reference				Reference				Reference				Reference		
E. coli (ESBL)	13.945	13.298	14.624	<0.001	4.596	4.399	4.801	<0.001	**Excluded (n=9337)*				**Excluded (n=9337)*				**Excluded (n=9337)*			
other *ESBL*	11.259	10.084	12.571	<0.001	12.772	11.310	14.423	<0.001	**Excluded (n=1463)*				**Excluded (n=1463)*				**Excluded (n=1463)*			
Klebsiella spp.	0.309	0.288	0.332	<0.001	0.301	0.284	0.318	<0.001	0.000	0.000	0.000	<0.001	1.276	1.218	1.335	<0.001	3.187	3.001	3.384	<0.001
Enterobacter spp.	0.440	0.388	0.500	<0.001	0.384	0.343	0.430	<0.001	53.457	43.926	65.056	<0.001	###	856.9	1914.5	<0.001	35.127	32.602	37.849	<0.001
Serratia spp.	0.596	0.487	0.729	<0.001	0.186	0.140	0.248	<0.001	75.764	48.104	119.3	<0.001	755.5	405.0	1409.4	<0.001	2110.7	1219.181	3654.310	<0.001
Citrobacter spp.	0.343	0.298	0.396	<0.001	0.230	0.201	0.265	<0.001	*Excluded (n=4056)				5.376	5.025	5.751	<0.001	8.371	7.693	9.110	<0.001
Proteus spp.	1.187	1.121	1.257	<0.001	1.914	1.830	2.003	<0.001	0.000	0.000	0.000	<0.001	0.537	0.495	0.582	<0.001	2.129	1.956	2.317	<0.001
Morganella spp.	0.891	0.748	1.061	0.196	1.189	1.032	1.370	0.016	*Excluded (n=1115)				*Excluded (n=1115)				254.7	207.865	311.968	<0.001
Providencia spp.	0.735	0.530	1.018	0.064	0.571	0.415	0.784	0.001	*Excluded (n=344)				*Excluded (n=344)				6.475	4.958	8.457	<0.001
others	0.658	0.488	0.887	0.006	0.610	0.474	0.786	<0.001	3.412	2.842	4.097	<0.001	4.767	3.994	5.690	<0.001	9.698	7.931	11.859	<0.001
**Postal code area (first digit)**				0.001				<0.001				<0.001				0.069				0.010
postcode area 5 (NRW)		Reference				Reference				Reference				Reference				Reference		
postcode area 2	0.890	0.840	0.942	<0.001	0.995	0.949	1.043	0.834	0.928	0.888	0.970	0.001	1.013	0.954	1.075	0.680	1.118	1.034	1.209	0.005
postcode area 3	1.064	0.883	1.282	0.517	1.313	1.140	1.513	0.000	1.278	1.119	1.460	<0.001	1.170	0.959	1.426	0.121	1.059	0.788	1.422	0.705
postcode area 4	1.000	0.962	1.039	1.000	1.091	1.057	1.126	<0.001	1.108	1.076	1.141	<0.001	1.062	1.018	1.107	0.006	1.087	1.025	1.152	0.005
postcode area 1	1.325	0.938	1.872	0.110	1.163	0.886	1.526	0.277	0.988	0.769	1.270	0.924	0.918	0.603	1.399	0.691	1.272	0.729	2.221	0.397
postcode area 6	0.813	0.582	1.136	0.225	0.882	0.682	1.139	0.335	0.965	0.774	1.204	0.752	0.912	0.641	1.298	0.608	0.771	0.442	1.345	0.359

CI: confidence interval; ESBL: extended-spectrum ß-lactamase; ICU: intensive care unit; OR: odds ratio.

[a] Very high intrinsic resistance rates in this species group did not allow the regression model to converge. Species groups with resistance rates of 100% to a specific antibiotic were therefore excluded from the respective regression analysis.

Multiple logistic regression model: Variable(s) entered: study year, sex, age, type of urine, healthcare setting, species groups, postcode area.

Supplementary Table S7: Resistance rates of ESBL-producing *E. coli* over time, Germany, 2016–2021 (n = 162268)

		E coli (ESBL)																				
		PIV			CIP			NIT			TRI			SXT			NF			FOS		
		Resistant		Total	Resistant		Total	Resistant		Total	Resistant		Total	Resistant		Total	Resistant		Total	Resistant		Total
		n	%	(n)	n	%	(n)	n	%	(n)	n	%	(n)	n	%	(n)	n	%	(n)	n	%	(n)
Study year	2016	NA		0	916	72.1%	1271	NA		0	751	59.6%	1260	706	55.4%	1274	54	4.2%	1271	46	3.6%	1269
	2017			0	1169	70.5%	1659			0	963	59.4%	1622	917	55.3%	1658	63	3.8%	1659	54	3.3%	1654
	2018			0	1275	65.0%	1961	0	0.0%	8	1147	59.4%	1932	1073	54.7%	1960	78	4.0%	1961	54	2.8%	1957
	2019	424	23.2%	1829	1192	62.7%	1900	0	0.0%	17	1053	55.5%	1899	966	50.8%	1901	75	3.9%	1901	49	2.6%	1901
	2020	356	20.9%	1705	1078	63.1%	1709	0	0.0%	28	952	55.9%	1704	841	49.2%	1709	68	4.0%	1710	40	2.3%	1710
	2021	141	17.0%	830	496	59.8%	830	0	0.0%	18	456	55.1%	827	412	49.6%	830	33	4.0%	830	17	2.0%	830

CIP: ciprofloxacin; FOS: fosfomycin; NF: nitrofurantoin; NIT: nitroxolin; PIV: pivmecillinam; SXT: trimethoprim/sulfamethoxazole; TRI: trimethoprim; n (r): number of resistant isolates; NA: not applicable; ESBL: extended-spectrum ß-lactamase;
